# Electrochemical CO_2_ reduction - The macroscopic world of electrode design, reactor concepts & economic aspects

**DOI:** 10.1016/j.isci.2022.104011

**Published:** 2022-03-04

**Authors:** Alina Gawel, Theresa Jaster, Daniel Siegmund, Johannes Holzmann, Heiko Lohmann, Elias Klemm, Ulf-Peter Apfel

**Affiliations:** 1 Department of Energy, Fraunhofer Institute for Environmental, Safety, and Energy Technology UMSICHT, Osterfelder Str. 3, 46047 Oberhausen, Germany; 2 Inorganic Chemistry I, Ruhr University Bochum, Universitätsstr. 150, 44801 Bochum, Germany; 3 Institute of Chemical Technology, University of Stuttgart, Pfaffenwaldring 55, 70569 Stuttgart, Germany

**Keywords:** Catalysis, Electrochemistry, Economics

## Abstract

For the efficient electrochemical conversion of CO_2_ into valuable chemical feedstocks, a well-coordinated interaction of all electrolyzer compartments is required. In addition to the catalyst, whose role is described in detail in the part “Electrochemical CO_2_ Reduction toward Multicarbon Alcohols - The Microscopic World of Catalysts & Process Conditions” of this divided review, the general cell setups, design and manufacture of the electrodes, membranes used, and process parameters must be optimally matched. The authors' goal is to provide a comprehensive review of the current literature on how these aspects affect the overall performance of CO_2_ electrolysis. To be economically competitive as an overall process, the framework conditions, *i*.*e*., CO_2_ supply and reaction product treatment must also be considered. If the key indicators for current density, selectivity, cell voltage, and lifetime of a CO_2_ electrolyzer mentioned in the techno-economic consideration of this review are met, electrochemical CO_2_ reduction can make a valuable contribution to the creation of closed carbon cycles and to a sustainable energy economy.

## Graphical abstract

**Figure:**
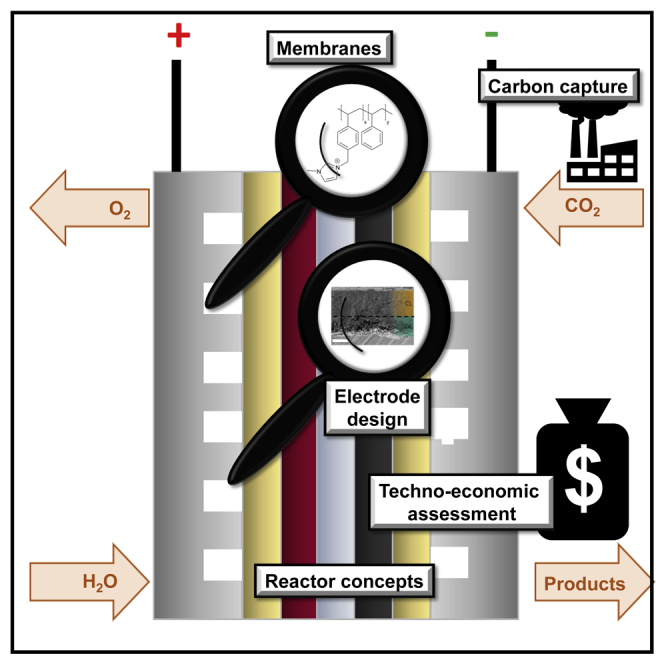

## Introduction

The electrochemical CO_2_ reduction reaction (CO_2_RR) powered by electrical energy from renewable sources offers encouraging potential to help counteract a further increase of atmospheric CO_2_ concentration and its adverse effects on planet Earth’s climate, ecosystems, and human health. Furthermore, it is providing an alternative to fossil resources as a carbon source. A variety of high-quality reviews on the topic have been published, covering specific catalysts and products, membranes, cell setups *etc* (Balamurugan et al., 2020; Fan et al., 2020; Hernandez-Aldave and Andreoli, 2020; Kibria et al., 2019; Kumar et al., 2016; Lu and Jiao, 2016; Nitopi et al., 2019; Rabiee et al., 2021; Salvatore et al., 2021; Weekes et al., 2018). However, scientific progress on the topic is rushing over the last years, electrochemical CO_2_ reduction is a process of high complexity, and the success of this technology is simultaneously subject to a multitude of factors.

One of those factors is the ambivalent role of water, which, on the one hand, is the source of protons necessary for CO_2_RR, and on the other hand, simultaneously a competing substrate for reduction in the hydrogen evolution reaction (HER). Maintaining the right balance and ensuring suitable mass transport of the reactants to and products from the electrode thus plays a key role in the cathodic process of CO_2_ electrolysis. In addition, ensuring sufficient quality and concentration of the CO_2_ feed before electrolysis as well as the separation of the diverse possible reduction products of CO_2_ after the actual electrolysis further influence the economic feasibility of the overall process and thus must be considered. To reduce costs in conjunction with product separation, the selective production of a single and concentrated product is highly desirable. As a measure for selectivity, the faradaic efficiency (FE) is commonly indicated, as it describes the percentage of electrons utilized for the formation of a particular product. Furthermore, any industrial implementation of CO_2_RR in the future will require a certain throughput to be economically competitive, which is corresponding to a partial current density of roughly 300 mA cm^−2^ reached for a specific product. In addition to the selectivity as one key criterion, an efficient electrolysis process thereby requires operation with low overvoltages to ensure optimal energy efficiency, which is benchmarked by overall cell voltages below 2 V (Jouny et al., 2018; Kibria et al., 2019). The derivation of those key performance indicators (KPIs) for an industrially feasible process will be described and discussed in the frame of the techno-economic assessment chapter of this review.

In conclusion, there is no doubt that besides the actual catalyst, the electrode and general reactor design significantly influence the performance of CO_2_ electrolysis regarding selectivity, energy efficiency, and profitability. Therefore, the following review focuses on technical aspects of the realization of the CO_2_ electrolysis including the description of different electrolyzer-types (H-type and flow cells), membranes, and product separation techniques (Burdyny and Smith, 2019). The development of stable, abundant, and cost-effective catalysts is of no lesser importance to achieve an adequate selectivity and feasible overpotentials in CO_2_RR, and quite the contrary requires extensive consideration for itself. Therefore, the first part “Electrochemical CO_2_ Reduction toward Multicarbon Alcohols - The Microscopic World of Catalysts & Process Conditions“ of this review concentrates on the role of the catalyst in electrochemical CO_2_ reduction. Hereby, we aim at painting a preferably comprehensive picture of the overall process and give an overview of the complexity of factors influencing the performance of electrochemical CO_2_ reduction.

It should be noted that all current densities given in this work are cathodic. For better comprehension, the authors refer to the absolute values, *i*.*e*. when discussing higher current densities, more cathodic values are meant.

## Overview: Compartments & reactor designs for CO_2_ electrolyzers

The research landscape on electrochemical CO_2_ reduction is vast and diverse, and the designs and operation modes of CO_2_ electrolyzers described in literature are just the same. The design of the electrochemical cell as well as the chosen compounds are of crucial importance for an efficient CO_2_RR performance.

**Figure 1:**
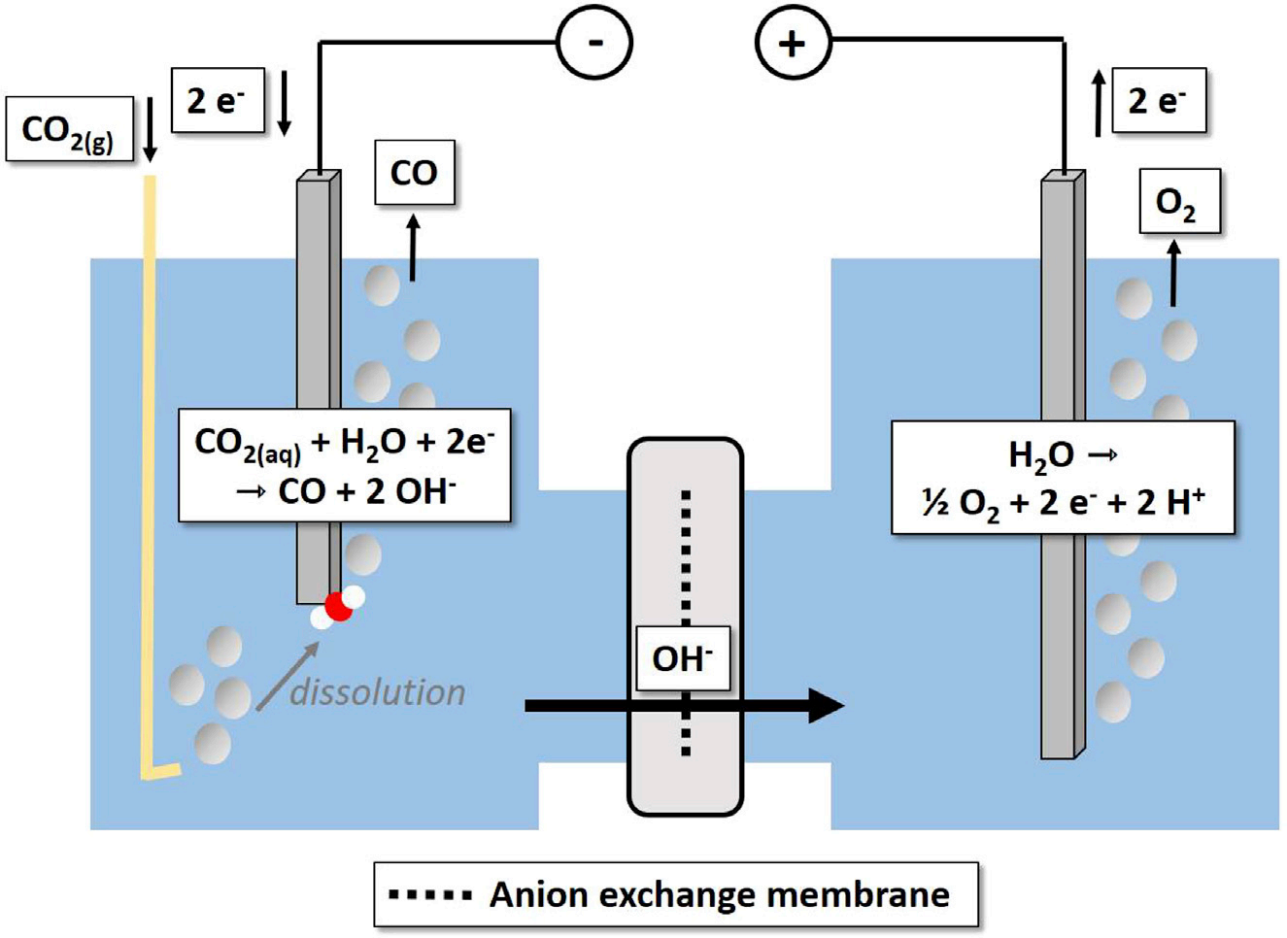
Schematic & simplified representation of fundamental processes taking place during CO_2_ electrolysis

However, the general processes taking place during CO_2_ electrolysis are illustrated in Figure 1. CO_2_ is supplied to the cathode in dissolved form. There, it is reduced to OH^−^ and, in our example, CO. Cathode and anode compartment are separated by an ion-conductive membrane, through which ion transport closes the electric circuit. In the illustrated case, an anion exchange membrane is used and OH^−^ migrates from the catholyte to the anolyte. At the anode water is oxidized to O_2_.

Fundamentally, electrolyzers can be subdivided into so-called H-type and flow cells, whereby in the latter case a distinction can be made between liquid- and solid-phase systems, depending on the physical state of the electrolyte (Kibria et al., 2019). The use cases, benefits, and limitations of those cells will be described in the following (chapters 3–5). However, the chapter will start with an overview of the currently used ion-exchange membranes as a component both H-type and flow cells have in common. Thereby, it is distinguished between proton, anion exchange, and bipolar membranes. As the membrane choice will be discussed in the context of all electrolyzer setups, we consider it appropriate to introduce them first.

In chapter 6, the role of CO_2_RR product separation is illustrated, as it is an important factor contributing to the profitability of CO_2_ electrolysis. The techno-economic assessment is then deepened in chapter 7. In chapter 8, we give an outlook on currently upcoming research topics in the field of electrochemical CO_2_ reduction.

## Ion-exchange membranes

Hardly any CO_2_ electrolyzer setup gets along without the implementation of ion-exchange membranes (IEM), which are necessary to limit product reoxidation and to allow for an effective separation of reaction environments in both half-cells (Zhang et al., 2020b). At the same time, IEMs must be able to close the electric circuit, *i*.*e*. to enable the passage of ions. By significantly contributing to the overall cell voltage, membranes also largely influence the total energy efficiency of the cell (Salvatore and Berlinguette, 2020).

**Figure 2:**
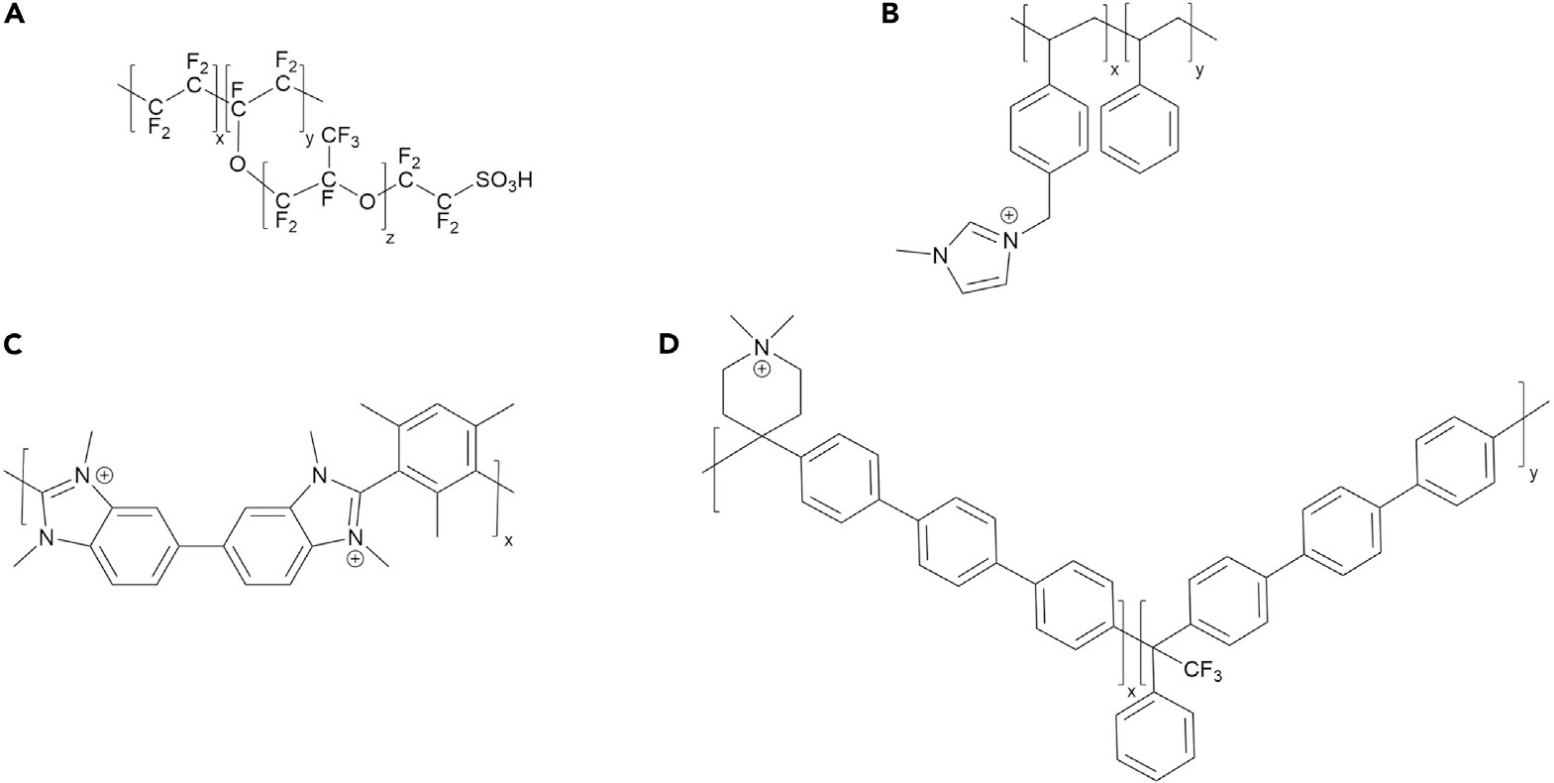
Chemical structures of common membrane ionomers (A) Nafion; (B) Sustainion; (C) Aemion; (D) PiperION (Endrődi et al., 2020; Kutz et al., 2017; Mauritz and Moore, 2004; Thomas et al., 2012).

A prominent example of a conductive polymer used to produce IEMs is Nafion (Figure 2A), which can be selectively crossed by cations and is therefore, in the context of electrolysis, categorized as a proton exchange membrane (PEM) (Albo et al., 2019; Han et al., 2020a; Lei et al., 2020; Martić et al., 2019; Song et al., 2020; Zhou et al., 2018).

However, the membranes used primarily for CO_2_ electrolysis are anion exchange membranes (AEMs) (Gao et al., 2018; García de Arquer et al., 2020; Hoang et al., 2018; Jeong et al., 2020; Karapinar et al., 2019; Kibria et al., 2018; Kibria et al., 2019; Li et al., 2019; Li et al., 2020; Lv et al., 2018a; Lv et al., 2018b; Ting et al., 2020; Wei et al., 2020; Yang et al., 2020b; Zhuang et al., 2018). A third type of conductive membranes is the bipolar membrane (BPM), which is a combination of a PEM with an AEM.

### Proton exchange membranes (PEMs)

Water oxidation, which is the most common anode reaction for CO_2_ electrolysis applications, leads to the formation of protons, whose flow to the cathode is enabled by a PEM (Weekes et al., 2018). Compared to AEMs, this electroosmotic flow toward the cathode inhibits product crossover to the anode. However, proton migration from the anolyte leads to a decrease of the pH of the catholyte, creating an environment at the cathode that favors HER over CO_2_RR and thus impeding to reach high FEs for CO_2_RR products. For that reason, in most instances, AEMs are employed for CO_2_ electrolysis.

Still, PEMs find use in three-compartment cells especially designed for the production of anionic liquid products, like formate or acetate. Thereby, anolyte and catholyte are not only separated by a single AEM, but by an additional central flow compartment and a PEM facing the anolyte. The central flow compartment is filled with a porous solid electrolyte to ensure both electrical contacting and product removal (Xia et al., 2019). In this setup, the reoxidation of generated liquid products because of crossover to the anode is prevented. The use of thicker membranes thereby significantly contributes to minimizing the product crossover from the central to the anode compartment, as shown by Masel and coworkers. Using the thinnest membrane, Nafion 212 (50.8 μm), the highest crossover of formate from the central flow compartment to the anolyte was observed. However, the use of thicker membranes also leads to increased electrical resistances and thus higher overall cell voltages. The lowest formate migration was obtained for Nafion 324 with a thickness of 150 μm. The authors attributed the lowered formate flux not only to the increased membrane thickness, but also to its structure, as it is a double-layered membrane consisting of two layers with equivalent weights (EW) of 1100 and 1500. Thereby, the equivalent weight describes the dry polymer mass per mole of acid groups. Masel and colleagues observed a markedly low water content in the 1500 EW layer, which, in combination with the decreased number of acid groups, provides unfavorable conditions for formate transport (Yang et al., 2017).

### Anion exchange membranes (AEMs)

Contrarily to a PEM, an AEM regulates the flow of anions from the cathode to the anode (Weekes et al., 2018). As the main charge carriers in CO_2_ electrolysis are carbonate and bicarbonate ions, AEMs provide lower polarization losses and thus higher limiting current densities compared to PEMs (Singh et al., 2015). To date, the most common examples of AEMs are made from Sustainion (Figure 2B). Sustainion membranes are stable toward bases and consist of a polystyrene backbone functionalized with imidazolium groups (Kaczur et al., 2018; Kutz et al., 2017; Liu et al., 2018; Yang et al., 2017). Before use, Sustainion and other AEMs are generally converted from chloride into the hydroxide form by storage in KOH (Kaczur et al., 2018; Liu et al., 2018). Thereby, the duration of this activation step can significantly influence the membranes' performance and has to be optimized (Ehelebe et al., 2020). Further modification of Sustainion membranes can lead to increased mechanical stability, as shown by Masel and colleagues. Employing a Sustainion membrane modified with divinylbenzene, they were able to operate an electrochemical cell for CO production stably for 3,800 h (158 days) at 200 mA cm^−2^ and 4 V (Liu et al., 2018). An upcoming alternative to Sustainion represents the benzimidazolium-based ionomer Aemion (Figure 2C) (Thomas et al., 2012). As Sustainion membranes are soluble, and thus unstable, in systems containing high ethanol concentrations, Sinton and coworkers employed an Aemion membrane for their ethanol-producing electrolyzer (Gabardo et al., 2019). Endrődi, Janáky, and colleagues reported the use of an AEM made from PiperION (Figure 2D), a poly(aryl piperidinium) structure characterized by high carbonate conductivity and mechanical robustness. Thereby, the authors achieved partial current densities for CO of >1 A cm^−2^ and a selectivity of up to 90% (Endrődi et al., 2020). Research on novel materials for AEMs is still ongoing, with the goal of finding highly conductive, robust, and cost-effective candidates to meet the growing demand.

### Crossover

Especially when using AEMs, the crossover of CO_2_ to the anode compartment plays an important role and lowers CO_2_ utilization, and is therefore associated with the overall efficiency of the cell (Weekes et al., 2018). The basic problem leading to the crossover of CO_2_ through the AEM is the reaction of CO_2_ with OH^−^ to carbonate (CO_3_^2−^) and bicarbonate (HCO_3_^−^) in the cathode compartment (Larrazábal et al., 2019; Lin et al., 2019; Liu et al., 2018; Pătru et al., 2019; Reinisch et al., 2019). Subsequently, OH^−^ in the membrane is exchanged with these carbonate species (Liu et al., 2018; Pătru et al., 2019). For every two electrons transferred in the electrolysis, crossover of two bicarbonate ions or one carbonate ion as current carriers through the membrane occurs (Pătru et al., 2019). The predominance of carbonate or bicarbonatein the catholyte depends on various aspects, such as mass transfer within the electrolyte, CO_2_ availability at the cathode, and the OH^−^ formation rate (Reinisch et al., 2019). In systems with pronounced HER at the cathode, a decrease in the CO_2_/O_2_ ratio at the anode becomes visible, presumably because of the crossover of OH^−^ formed in the HER instead of carbonate species (Larrazábal et al., 2019). The anions transported through the AEM are reoxidized to CO_2_ in the anode chamber according to 2CO_3_^2−^ → 2 CO_2_ + O_2_ + 2 e^−^ (Larrazábal et al., 2019; Pătru et al., 2019). The preferential oxidation of carbonate toward water was confirmed by the observation that, at high current densities, twice as much CO_2_ as O_2_ is formed at the anode (Pătru et al., 2019). Overall, crossover of CO_2_ through the membrane is influenced by various factors, which includes membrane chemistry, electrolyte composition, and mass transfer of CO_2_ to the reaction interface (Larrazábal et al., 2019). The overpotential at the cathode has no influence on the transport of CO_2_ through the membrane, as the same amount of OH^−^ per reduction equivalent is formed independently from the selectivity of the reaction. CO_2_ utilization can be improved by the addition of buffers to the electrolyte, as the buffer anions act as alternative current carriers through the AEM (Lin et al., 2019).

Similarly to CO_2_ crossover, also the products of the CO_2_RR can migrate through an AEM (Zhang et al., 2020b). Thereby, charged products, such as acetate and formate, migrate more frequently into the anode compartment than uncharged products, e.g., ethanol and propanol, because of their role as current carriers. This so-called electromigration is observed across all current density ranges (Ma et al., 2020a; Zhang et al., 2020b). Thus, crossover of both CO_2_ and its reduction products to the anode compartment and their subsequent reoxidation limit the overall efficiency and CO_2_ utilization when AEMs are used in CO_2_ electrolyzers (Pătru et al., 2019).

### Bipolar membranes (BPMs)

**Figure 3:**
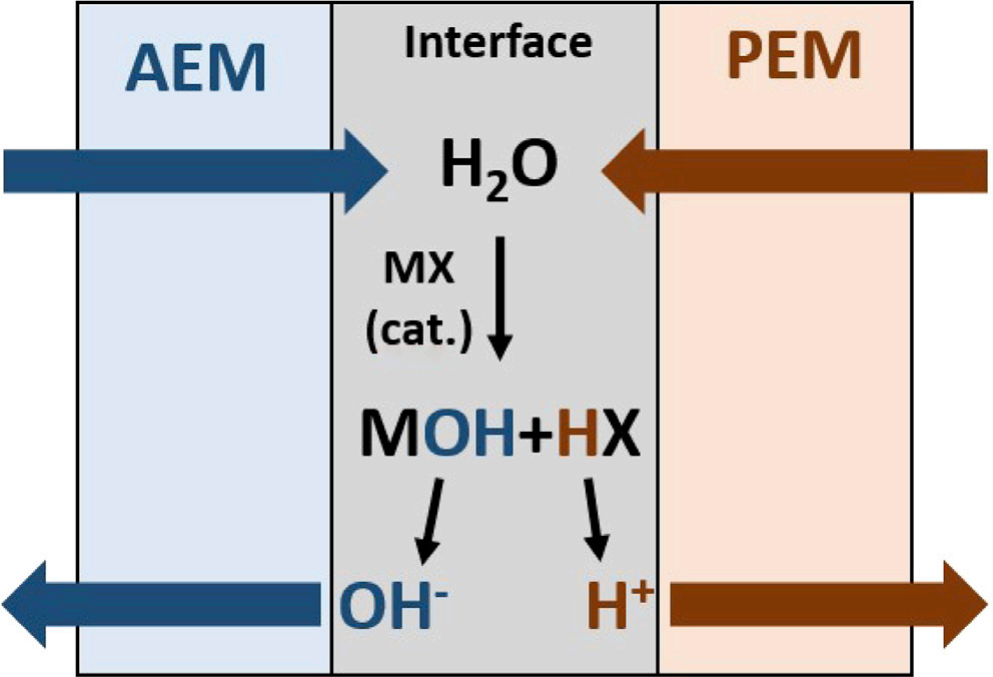
Schematic drawing of a bipolar membrane

As an alternative to AEMs with the goal to minimize product crossover, bipolar membranes (BPMs) are employed in CO_2_ electrolysis. BPMs are produced by the lamination of a PEM and an AEM (Figure 3). Thereby, strong ionic functional groups of the membranes themselves, e.g. sulphonic or carboxylic groups in PEMs or amine or imidazole groups in AEMs, often help to facilitate water dissociation. Alternatively, water dissociation catalysts can be added to the membranes' interface, like ionic polymers or metal oxides/hydroxides, salts or complexes (Pärnamäe et al., 2021).

In BPMs, product crossover is prevented because instead of ion flow from one electrode to the other, water dissociates into OH^−^ and H^+^ in the BPM connection layer and is transported through a PEM in the direction of the cathode and through an AEM to the anode, respectively (Li et al., 2016; Salvatore et al., 2018). Still, crossover of ions through a BPM by both diffusion (at low current densities up to 40 mA cm^−2^) and electromigration take place to a certain extent. Lowest relative ion crossover is observed at high current densities independently from the electrolyte (Blommaert et al., 2020). Research efforts are still needed to increase the durability of BPM and decrease their through-plane resistance. However, the voltage drop caused by water splitting is inevitable and can at best be reduced by means of suitable catalysts (Blommaert et al., 2019).

### Direct comparison of different membrane types in CO_2_RR

Table 1 gives an overview of the advantages and disadvantages of the different membranes for their application in CO_2_RR. Along the line of direct comparisons of different membrane types in the context of CO_2_RR, Xiang and colleagues compared an AEM (Fumasep AA-3-50; 45–55 μm), a PEM (Nafion 324; 150 μm), and a BPM (Fumasep FBM; 130–160 μm) with a focus on CO_2_ utilization of the different systems. AEM and BPM showed stable cell voltages during the measurements, whereas conductivity loss of the anolyte led to continuously increasing cell voltages when the PEM was used. Circumventing cation depletion in the anolyte by recirculation and mixing of the catholyte and anolyte resulted in a CO_2_ utilization efficiency of 15.1% for the PEM-based system, which is comparable to the value obtained for the AEM-based reactor (14.4%). The by far highest CO_2_ utilization efficiency, ranging from 58.0–61.4%, was obtained using the BPM-based reactor (Lin et al., 2019). Conversely, in another comparison of different membranes, Seger and coworkers found that for the Fumasep FAA-3-PK-75 (AEM), the Nafion 212 (PEM), and the Fumasep FBM (BPM) membranes, despite their different operating principles, the CO_2_ consumption measured in 1 M KHCO_3_ and at 200 mA cm^−2^ was nearly the same. The authors point out that the majority (≥65%) of the CO_2_ consumption is attributed to carbonate formation in direct proximity of the cathode and that this process is nearly unaffected by the membrane selection. However, CO_2_ utilization was slightly higher in the BPM-based system: In contrast to the AEM, carbonate does not migrate to the anolyte. Likewise, H^+^ ions migrating to the catholyte from the BPM led to the rerelease of CO_2_, making it again available to the reaction. The PEM did not offer this benefit, as K^+^ was found to be the main charge carrier. Furthermore, also the FEs measured did not depend on the type of membrane used, indicating that herein the membrane selection was not relevant for the observed catalytic selectivity. This observation supports the hypothesis that the direct environment of the cathode is hardly influenced by the utilized membrane. However, bulk catholyte pH increased from 8.3 to 9.8 using the PEM, 10.2 using the AEM, and <9 using the BPM, respectively. Because of anolyte conductivity loss, the PEM-based system was not suitable for long-term (>3 h) operation. Furthermore, the authors reported that product crossover was similar for charged and uncharged products but quantitatively negligible in case of the BPM and PEM. For the AEM, crossover of formate and acetate to the anolyte was found (Ma et al., 2020b).

**Table 1:** Comparison of advantages and disadvantages of PEM, AEM & BPM in CO_2_RR

Membrane type	Advantages	Disadvantages
PEM	- cost-effective because of simple manufacturing processes (Ramdin et al., 2019) - low voltage drop because of thinner membrane (Ramdin et al., 2019) - high stability results in increased lifetime (Ramdin et al., 2019) - used in three-compartment cells for the formation of liquid ionic products (Xia et al., 2019; Yang et al., 2017)	- low pH at the cathode suppresses CO_2_RR and favors HER (Pătru et al., 2019) - higher product crossover than BPM - expensive purification steps of electrolyte necessary (Ramdin et al., 2019) - not always resistant to oxidation reactions (Yang et al., 2017)
AEM	- no delivery of H^+^ to cathode, ensuring a high pH value at the cathode and thus creating favorable conditions for CO_2_RR over HER (Weekes et al., 2018) - more cost-effective than BPM (Weekes et al., 2018) - stable cell voltages during electrolysis (Lin et al., 2019) Sustainion - highly conductive (Kutz et al., 2017; Liu et al., 2018; Yang et al., 2017) - stable against alkaline electrolytes (Kaczur et al., 2018; Yang et al., 2017) - high ion exchange capacity (Kaczur et al., 2018) - 1,000–3,000 h stable during process (Kaczur et al., 2018) - functionalization possible (Yang et al., 2017)	- CO_2_ crossover to anode compartment (Larrazábal et al., 2019; Lin et al., 2019; Pătru et al., 2019; Reinisch et al., 2019; Weekes et al., 2018) - outgassing of CO_2_ at anode (Ma et al., 2020a; Ma et al., 2020b) - in part crossover of liquid products (EtOH, *n*-PrOH) (Ma et al., 2020a) - use of conc. KOH can lead to precipitation of K_2_CO_3_ ⇾ decrease in current density (Endrődi et al., 2019) Sustainion - unstable at high EtOH concentrations (Gabardo et al., 2019)
BPM	- PEM side inhibits CO_2_ transport into the anode compartment (Pătru et al., 2019) - constant pH gradient (Ramdin et al., 2019; Weekes et al., 2018) - low product crossover/product loss (Ramdin et al., 2019) - acid/base addition for acidification/basification possible (Ramdin et al., 2019) - stable cell voltages during electrolysis (Lin et al., 2019)	- complex and expensive production (Ramdin et al., 2019) - low stability of AEM especially in alkaline medium (Ramdin et al., 2019) - short lifetime because of delamination of the ion exchange layers (Ramdin et al., 2019)

An important property of membranes is their high conductivity, which is necessary to ensure low operation voltages and thus high energy efficiency. In one example using KCl and K_2_SO_4_ as electrolytes, examination of a Fumatech Nafion PEM and FAA-3 AEM showed that operation at elevated current densities (>200 mA cm^−2^) resulted in overvoltages that led to system overload in both cases. As the reason for this overvoltage, the authors stated high membrane resistance caused by insufficient ionic conductivity. Therefore, the necessity to develop either suitable membranes or membrane-free cells is pointed out (Lv et al., 2018a). In the context of the development of such high-performance conductive membranes, Masel and coworkers compared the area-specific resistance of Sustainion 37–50 with other commercially available membranes. With a value of 0.045 Ω cm^−2^ in 1 M KOH at 60°C, the resistance was more than one order of magnitude lower compared to the resistances of other membranes, such as Nafion N115 (0.52 Ω cm^−2^), Fumasep FAPQ-375 (0.83 Ω cm^−2^) or PBI (phosphoric acid doped polybenzimidazole, 8.3 Ω cm^−2^) (Kaczur et al., 2018). Regarding the influence of the membrane on the ratio of HER and CO_2_RR, a Sustainion PSMIM (polystyrene methyl methylimidazolium chloride) AEM was compared with various PEMs (Nafion 117, CMI-7000, SPEEK (sulfonated poly(ether ether ketone)), and PBI (polybenzimidazole)) and other AEMs (Neosepta, AMI-7001, PVA (polyvinyl alcohol), and PEI (polyethylenimine)) (Kutz et al., 2017). Therefore, electrochemical tests were performed with moistened CO_2_ as water supply, because liquid electrolytes based on KHCO_3_ would poison the acidic PEMs and KOH could leach some of the AEMs. Because Ag was used as a cathode catalyst, CO and H_2_ were obtained as the only reduction products. The selectivity for the target product CO was lowest for the PEMs, whereby the imidazolium-containing variant PBI performed slightly better (Nafion 117 ≈ CMI-700, 0% < SPEEK, < 10% < PBI, < 20%). Among the AEMs, selectivity grew from PEI (<20%) over AMI-7001 (<30%), Neosepta (<40%) and PVA (<60%) to PSMIM (>90%). Therefore, also among the AEMs, the imidazolium-doped variant PSMIM showed enhanced performance. This observation is in agreement with the results of other studies stating imidazolium to be a cocatalyst for CO_2_RR and contributing to HER suppression (Kutz et al., 2017; Rosen et al., 2013; Yang et al., 2017).

In summary, membranes used for electrochemical CO_2_ reduction have to provide distinct electric conductivity to minimize the overall cell voltage. AEMs mostly outperform PEMs by ensuring a sufficiently high catholyte pH, which helps favoring CO_2_RR over HER, and maintaining stable cell voltages by avoiding cation depletion in the anolyte. However, a drawback of AEMs is the possible crossover of CO_2_ and products to the anolyte. A promising approach to limit general crossover is the use of BPMs; however, BPMs are in turn prone to delamination. Functionalization of membranes with imidazolium can furthermore help to boost selectivity toward CO_2_RR. Because considerable progress has been made in the field of membranes in recent years, further substantial improvements regarding stability, conductivity or cost-effectiveness can be expected in the future.

## H-type cells - setup, use cases & benefits

**Figure 4:**
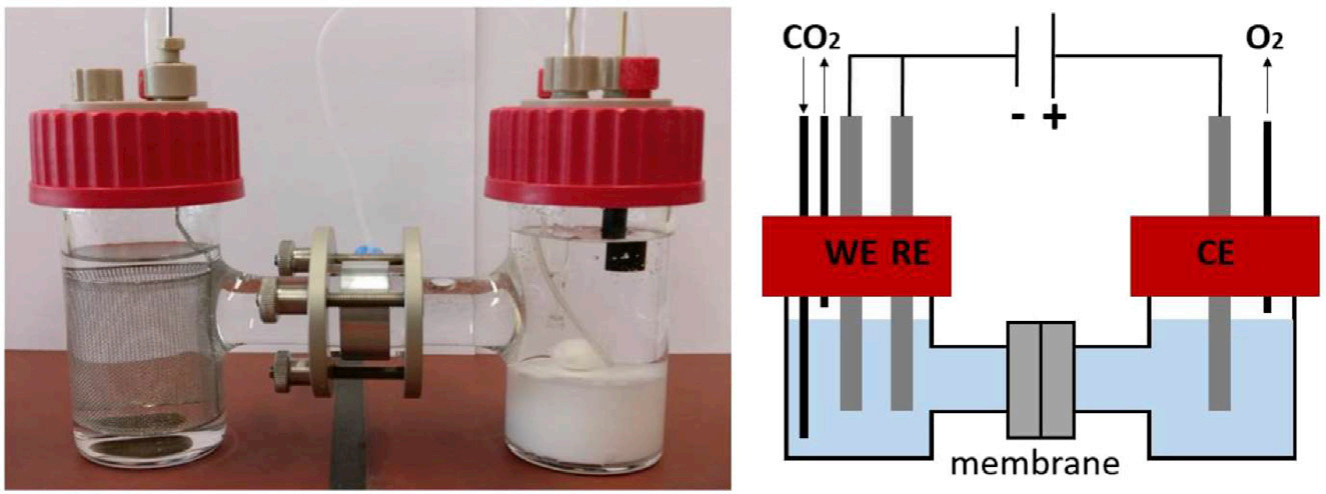
H-Type cell with a three-electrode arrangement for CO_2_ reduction Left: Picture of an exemplary laboratory-scale H-type cell. Right: Schematic drawing.

H-type cells are liquid-phase electrolyzers that are widespread in fundamental research on the electrochemical CO_2_ reduction (Dutta et al., 2020; Gao et al., 2018; Han et al., 2020a; Iijima et al., 2019; Jeong et al., 2020; Jiang et al., 2018b; Lei et al., 2020; Lv et al., 2018b; Shang et al., 2019; Song et al., 2020; Ting et al., 2020; Wei et al., 2020; Zhou et al., 2018). The name is derived from the H-like form (depicted in Figure 4) of the reactor with cathode and anode compartment filled with liquid electrolyte and separated by an ion exchange membrane to prevent the reoxidation of products. CO_2_ is supplied to the cathode via dissolution in the catholyte, which is why the transport of CO_2_ to the catalyst surface takes place exclusively through the electrolyte (Burdyny and Smith, 2019; García de Arquer et al., 2020; Xiang et al., 2019).

H-type cells are widespread particularly for studies that focus on catalyst design and screening, as the setup allows for simple and rapid testing. In terms of electrode preparation, the catalyst is usually deposited on glassy carbon or carbon paper, which is mainly done via electrodeposition, spin coating, dipcoating, or drop-coating (Cook, 1988; Dinh et al., 2018a; Dutta et al., 2020; He et al., 2017; Hou et al., 2020; Lamaison et al., 2020). Also, the use of bulk catalyst material (e.g. polycrystalline Cu) was reported (Ahn et al., 2017; Hori et al., 1986; Kaneco et al., 2002). Furthermore, we recently described the use of bulk pentlandite pellet electrodes for catalyst characterization in H-type cells (Pellumbi et al., 2020; Piontek et al., 2019).

The reaction conditions exert significant influence on the performance of CO_2_RR in an H-type cell. Here, the solubility of CO_2_ in any electrolyte is directly affected by temperature. Because of higher solubility and thus availability of CO_2_, reduced reaction temperatures have been shown to contribute to favoring CO_2_RR over HER (Ahn et al., 2017). On the other hand, elevated temperatures enhance the diffusion coefficient of CO_2_, contributing to its availability in the opposite way. In addition, the temperature influences the electric conductivity of the electrolyte. It is therefore suspected that, for each individual reaction system, temperature has to be optimized (Löwe et al., 2019). For instance, the necessity of temperature optimization was demonstrated by Palmore and colleagues. They studied the influence of the reaction temperature on electrochemical CO_2_ reduction using polycrystalline copper as catalyst and found different temperature optima, depending on the target product. Although the FE for methane increased with decreasing temperatures and peaked at 2°C, the FE for ethylene increased at elevated temperatures, reaching its maximum at 22°C (Ahn et al., 2017). This observation was confirmed in various studies and illustrates how the processes contributing to the selectivity of CO_2_RR products are influenced by temperature in different ways (Cook, 1988; Hori et al., 1986; Kim et al., 1988).

Apart from the temperature, also the pressure in an H-type cell influences CO_2_ solubility, and thus availability. Applying pressure to an H-type electrolyzer cell increases CO_2_ solubility and therefore also the achievable current densities. For example, although CO_2_ solubility in water at atmospheric conditions at 25°C is only 0.033 mol L^−1^, it can be increased to 1.17 mol L^−1^ by applying a pressure of 60.8 bar. Furthermore, changes in selectivity of the CO_2_RR were reported and attributed to facilitated CO desorption at higher CO_2_ pressures (Hori and Murata, 1990; Kibria et al., 2019; Kudo et al., 1993; Ramdin et al., 2019). A common observation is a reduction in FEs for HER and methane, assigned to increased coverage of the surface with CO (Hara, 1994; de Jesús-Cardona et al., 2001; Kas et al., 2015).

### Limitations

The use of H-type cells is limited to a set of specialized applications mainly in the field of catalyst characterization (Burdyny and Smith, 2019). Because the solubility of CO_2_ in aqueous electrolytes under atmospheric conditions is as low as 34 mM, electrolysis carried out in H-type cells are prone to reach mass-transport limitations and therefore limited in current density to values well below 100 mA cm^−2^ (Fan et al., 2020; Kibria et al., 2019; Malkhandi and Yeo, 2019; Weekes et al., 2018). Furthermore, the delivery of CO_2_ in dissolved form impedes the use of basic electrolytes, as a significant proportion of the dissolved CO_2_ reacts with OH^−^ to form carbonate (Malkhandi and Yeo, 2019). The consequences are not only a loss of CO_2_ but additionally a reduced CO_2_RR activity, a lower conductivity of the electrolyte, and a shift of the pH value toward a more acidic milieu (Carroll et al., 1991; Kibria et al., 2019). As the local conditions at and the selectivity of a CO_2_RR catalyst are highly dependent on the applied current density and potential, results obtained in an H-type cell are not merely transferable to its performance under industrially relevant conditions. Therefore, testing and optimization of catalysts under realistic conditions at higher current densities demands the use of alternative setups (Burdyny and Smith, 2019; Weekes et al., 2018). To overcome the limitations of the CO_2_ solubility in aqueous electrolytes, organic solvents such as acetonitrile or methanol are also commonly used in H-type cells. In case of aprotic solvents, traces of water, however, have to be added to enable any CO_2_ reduction. To increase conductivity, conducting salts, e.g. TBAPF_6_ (tetrabutylammonium hexafluorophosphate), are used. Obviously, it is problematic to use potential CO_2_ reduction products like alcohols as solvents, because precise FEs can no longer be determined. Furthermore, it has to be ensured that CO_2_ itself is the substrate for the reduction reaction and the observed products do not originate from undesired side reactions of the solvent. To clarify the origin of a reduction product, isotope-exchanged solvents or ^13^CO_2_ can be used (Oh et al., 2014; Pellumbi et al., 2020; Piontek et al., 2019). Notably, organic electrolytes are suitable for catalysts screening, characterization, and comparison but not for industrial application, as the reachable current densities are insufficient. Furthermore, the prize and the low environmental compatibility of most organic solvents hinder their large-scale use.

## Liquid-phase flow cells & gas diffusion electrodes

In order to achieve an economic operation of any electrolyzer device, a minimum current density of 200 mA cm^−2^ is frequently stated for CO_2_ reduction products in which two electrons are transferred. In case of multi-electron transfer, as in the formation of multicarbon products, these limits must be correspondingly higher to yield the respective molar amount per area (Burdyny and Smith, 2019). To operate CO_2_RR under those industrially relevant conditions, different versions of flow cells were developed. Generally in flow cells, the transport of CO_2_ to the catalyst takes place via the gas phase and is therefore not limited by the solubility of the gas in an electrolyte. Gas and electrolyte streams are continuously cycled, allowing for current densities well above 200 mA cm^−2^ and the potential stacking of electrolyzers in large scale applications (Li et al., 2019; Martić et al., 2019; Weekes et al., 2018). The reactor designs can mainly be categorized into liquid-phase electrolyzers and solid-phase electrolyzers, depending on the physical form of the electrolyte. High-temperature solid-oxide electrochemical cells (SOECs), where a ceramic solid electrolyte is used instead of a conductive polymer, represent a special application and therefore will also be briefly described (Kibria et al., 2019).

### Setup & anode

**Figure 5:**
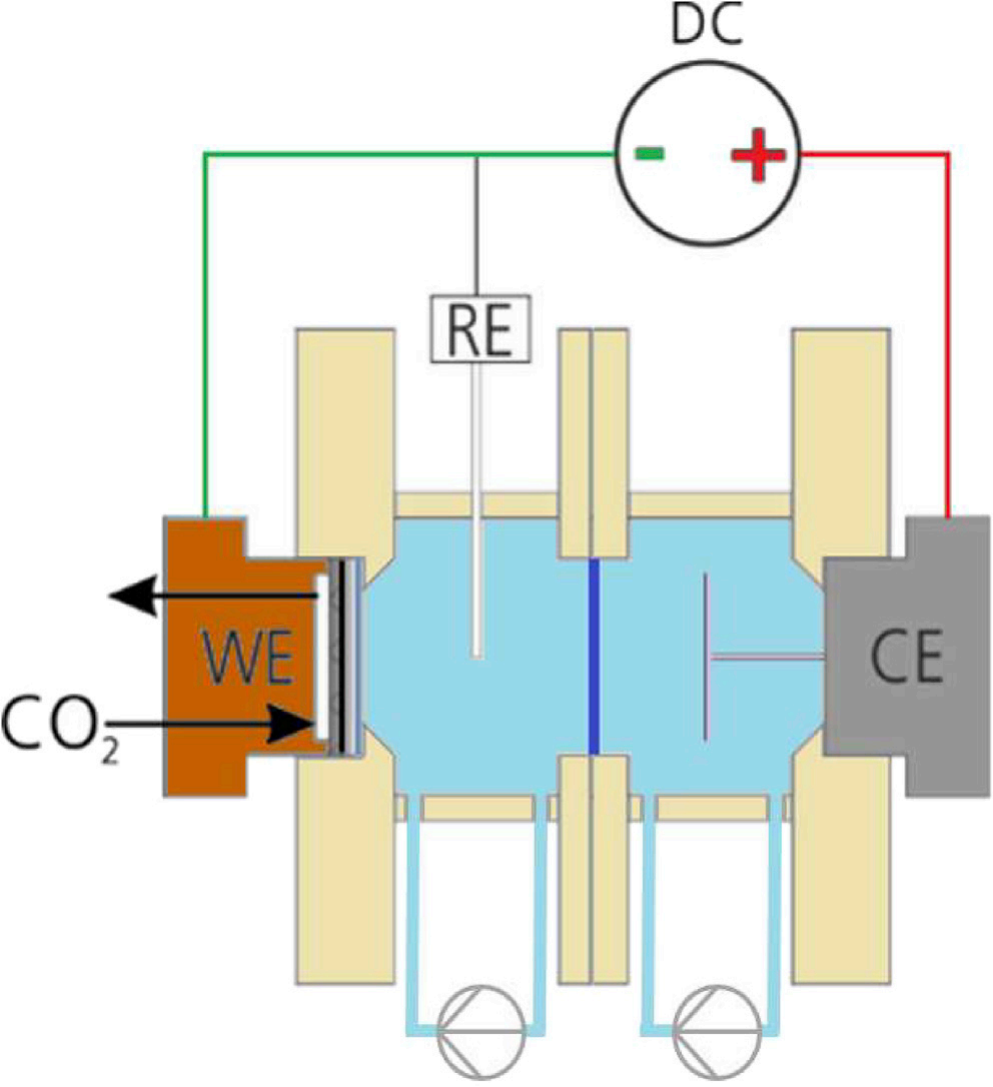
Liquid-phase electrolyzer with electrolyte circulation Schematic representation. WE: working electrode; RE: reference electrode; CE: counter electrode.

**Figure 6:**
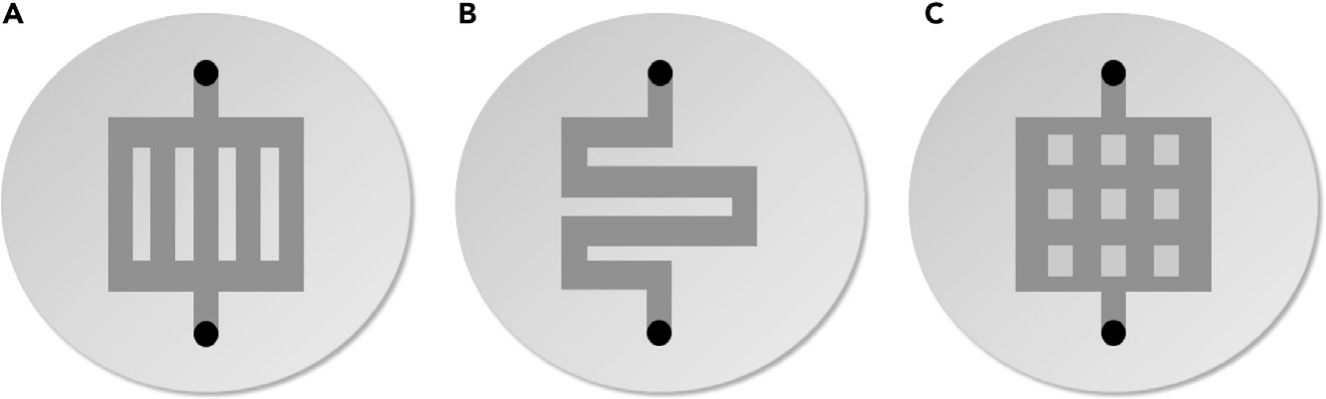
Flow field geometries (A–C) Schematic depiction of different flow field geometry types. (A) parallel geometry, (B) serpentine geometry, (C) pin geometry.

Figure 5 provides a schematic illustration of a liquid-phase flow cell architecture. Alternatively, a zero-gap arrangement between anode and IEM can be used to minimize ohmic losses. The zero-gap concept will be further described in the subsequent chapter. The cathodic and the anodic compartments are separated by an IEM to allow for the migration of ions while mitigating the crossover of desired liquid products to the anode side and of anode reaction products to the cathode site (Kibria et al., 2019; Weekes et al., 2018). Besides the membrane, the performance of a liquid-phase flow cell can also be influenced by the fluid dynamics. Therefore, flow fields of different geometries (Figure 6) are applied to direct both the gas and the electrolyte flow at the electrodes and function as electrical contact.

By ensuring a homogeneous current distribution, flow fields prevent the formation of efficiency gradients within the electrodes (Weekes et al., 2018). In the example of PEM fuel cells, it was shown that a large contact area between the gas diffusion electrode (GDE) and the flow field of the bipolar plate is beneficial to enable higher current densities. Furthermore, the current distribution in the GDE was more uniform for shorter-channeled flow fields; therefore, the use of serpentine-type and pin-type flow fields, unlike parallel flow fields, prevented the undesired formation of preferential flow paths, which lead to inhomogeneous reagent distribution. In the case of PEM water electrolyzers, it was found that the geometry of the cathode flow field largely influences the ohmic overpotential, whereas the anode flow field geometry was crucial for water availability (Jeanty et al., 2018; Li et al., 2018; Lin et al., 2020). However, the transferability of these results to CO_2_ electrolyzers is limited leading to a knowledge gap about the effects of flow field geometry on CO_2_RR.

While CO_2_ RR occurs at the cathode, oxygen evolution reaction (OER) is the primarily chosen counter reaction at the anode. The anode reaction also affects the overall energy efficiency of the cell. Hence, publications dealing with the optimization of the OER catalysis have been published (Jiang et al., 2018a; Ma et al., 2014). Also alternative anode reactions, such as the oxidation of chloride to chlorine, have been tested to improve the energy efficiency of the CO_2_ reduction cells (Kong et al., 2019; Lister and Dufek, 2013; Verma et al., 2019).

### Cathode: gas diffusion electrodes (GDEs)

In contrast to an H-type cell, the CO_2_ reduction reaction in a liquid-phase flow cell takes place at a gas diffusion electrode to overcome the issues concerning CO_2_ solubility and mass transfer limitations (Albo et al., 2019; Chen et al., 2020b; García de Arquer et al., 2020; Hoang et al., 2018; Karapinar et al., 2019; Kibria et al., 2018; Li et al., 2019; Li et al., 2020; Lv et al., 2018a; Martić et al., 2019; Ren et al., 2019; Xiang et al., 2019; Yang et al., 2020b; Zhuang et al., 2018).

GDEs allow for sufficient supply with CO_2_ even at higher current densities because of the reduced diffusion pathway, when CO_2_ is provided from the gas phase, compared with diffusion from CO_2_ dissolved in aqueous solution (Burdyny and Smith, 2019). The exact phase distribution and the extent of the reaction zone in GDEs is a matter of ongoing debate. Although multiple authors suggest that the reaction is taking place at the three-phase boundary between solid catalyst, liquid electrolyte, and gaseous CO_2_ supply, Smith and coworkers conclude that a two-phase reaction between the catalyst and CO_2_ dissolved in the electrolyte is more likely (Nesbitt et al., 2020). Either way, the processes taking place in a GDE are far more complex compared to those in a conventional H-type cell. Therefore, CO_2_RR at GDEs is prone to relatively small changes in wettability, texture, and morphology, which poses a challenge to reproducibility (Bohra et al., 2020). Furthermore, the complexity of the system makes it hard to predict the optimal composition and structure for a GDE.

**Figure 7:**
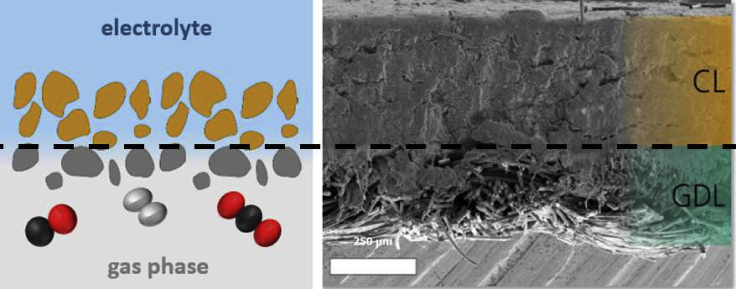
Cross section of a gas diffusion electrode Left: Schematic representation; catalyst layer particles are colored in brown and gas diffusion layer particles in gray, respectively. Right: Scanning electron microscopy (SEM) image showing the catalyst layer (CL) and the gas diffusion layer (GDL). Figure based on reference (Junge Puring et al., 2021).

Gas diffusion electrodes typically consist of a gas diffusion layer (GDL), which is mostly a dense network of carbon fibers such as carbon paper or carbon cloth or a polytetrafluoroethylene (PTFE) membrane and the catalyst layer (CL). Carbon-based GDLs are furthermore often coated with a microporous layer (MPL), usually consisting of compressed carbon powder or fibers and PTFE (Malkhandi and Yeo, 2019; Weekes et al., 2018). The GDE is installed in the cathode compartment of the flow cell with the catalyst layer facing the electrolyte and the support facing the gas side, where CO_2_ is supplied (Figure 7).

The material properties of GDEs are adjustable by the choice of individual components and the process of electrode manufacturing. Apparently, high electrical conductivity is of utmost importance for a GDE to operate with low ohmic overpotential. To modulate mass transport to the catalyst, both porosity and hydrophobicity are particularly relevant. A certain mechanical stability is furthermore helpful for simple incorporation into the cell setup and facilitated upscaling (Weekes et al., 2018).

### GDE fabrication

A variety of GDE fabrication methods was reported in literature including the deposition of the catalysts on or mixing it with carbon black as well as an additional binder like PTFE followed by hot pressing of the CL on a GDL (Cook, 1990; Junge Puring et al., 2021; Löwe et al., 2019). Alternatively, GDEs can also be obtained by airbrushing/spray coating or drop casting of a catalyst ink onto a GDL (Gregorio et al., 2020; Hara and Sakata, 1997; Leonard et al., 2020; Marcos-Madrazo et al., 2019; Martić et al., 2020; Merino-Garcia et al., 2018; Shafaque et al., 2020; Wang et al., 2019; Zhang et al., 2020c). Along this line, Berlinguette and coworkers examined the fabrication of GDEs by spray coating with a focus on a uniform distribution of the ionomer. They found significant deviations from the targeted Nafion contents on the electrode for every tested deposition method, with the most homogeneous results achieved by automated spray coating (Lees et al., 2020). Furthermore, the catalyst ink can also be painted on the GDL (Whipple et al., 2010; Xiang et al., 2019). In cases where a metal catalyst should be applied on a conductive, mostly carbon-based GDL, direct electrodeposition is another possibility for GDE fabrication (Henckel et al., 2021). In addition, atomic layer deposition (ALD) and sputtering of substrates onto GDLs are frequently reported (G. Dinh et al., 2018b; Ren et al., 2019; Tiwari et al., 2018; Wang et al., 2020b). The method of catalyst application strongly influences the catalyst loading and correspondingly the thickness of the CL, which are influential properties of a GDE. If the layer thickness is increased, the diffusional mass transfer resistance simultaneously increases and a modulation of the local CO_2_ and product concentration takes place. Modulating those concentration ratios exerts a marked influence on the selectivity of the CO_2_RR.

The local CO_2_ concentration at the GDE can also be varied directly by applying different CO_2_ feed flow rates, as reported by Oh and coworkers. They demonstrated that reducing the rate from 40 sccm to five sccm results in a remarkable increase of the selectivity of C_2+_ products (from FE = 44.9 to 60.3%), and decrease of C_1_ products, respectively (Tan et al., 2020). However, a higher catalyst loading does not necessarily lead to an improved selectivity of a GDE, as it has more implications than just the modulation of reactant concentrations. For instance, Neyerlin and colleagues observed the influence of CL thickness on the selectivity of a SnO_2_ GDE toward formate formation and obtained best selectivity at the lowest catalyst loading. They explained this observation with surplus catalyst-ionomer active sites, which favor, contrarily to catalyst-catholyte active sites, HER (Chen et al., 2020c). Besides the catalyst loading, also its structuring and the addition of various additives to the CL influence the properties and performance of a GDE. When examining different configurations of CLs based on Ag and multiwalled carbon nanotubes (MWCNTs), Kenis and coworkers found that a mixed layer of MWCNTs and Ag particles yielded the lowest charge-transfer resistance compared to a layered setup and bare Ag particles. Because the availability of the active catalyst sites was improved by the MWCNTs, twice as high current densities were obtained at half the loading for the mixed and layered versions compared to the GDE without MWCNTs (Ma et al., 2016a). However, addition of a layer of graphene oxide onto the CL of a GDE was shown to provide an effective barrier toward proton mass transport and thus led to markedly favored CO_2_RR over HER (from 40% FE_CO_ without any coverage to 80% FE_CO_ with 90% coverage) (Le Nguyen et al., 2020). A similar effect was obtained by Perry and coworkers, who applied a hydrophobic layer of 1-octadecanethiol onto a Cu-based GDE. Up to 100 mA cm^−2^, this layer markedly suppressed HER. However, at higher current densities, proton transport to the catalyst was insufficient and the performance was comparable to the untreated electrode (Perry et al., 2021). In general, the limitation of proton supply to the CL of a GDL is a promising approach to enhance FEs for CO_2_RR. Therefore, besides its role as a binder in thermally treated catalyst layers, PTFE is also used as an additive in spray-coated GDEs to modulate the catalyst's environment. Because of its hydrophobic properties, PTFE thereby prevents the CL pores from penetration of liquid electrolyte, potentially leading to favoring of CO_2_RR over HER. Feng and colleagues, for example, were able to increase the partial current density for CO_2_RR from below 150 mA cm^−2^ to approximately 250 mA cm^−2^ at −1.0 V vs. RHE by addition of 50% PTFE to the CL in the form of preferably small particles (30–40 nm). However, at higher PTFE percentages, the proton supply necessary for CO_2_RR was inhibited, resulting in again decreasing partial current densities (Xing et al., 2021). In addition, promising results regarding the suppression of HER and selectivity modulation for CO_2_RR were reported using surfactants, such as Triton X-100 (octyl phenol ethoxylate) or CTAC (cetyltrimethylammonium chloride). However, their use is hitherto only described as a surface coating for planar electrodes in an H-type cell or as an additive to the electrolyte in liquid-phase flow cells (Banerjee et al., 2019; Bienen et al., 2020; Zhong et al., 2020). Hence, direct modification of GDEs with surfactants still represents an open research topic.

### GDL materials

The reason why most GDLs used for CO_2_RR are based on carbon is, on the one hand, their outstanding conductivity and the uniform current distribution provided by this material (G. Dinh et al., 2018b). On the other hand, carbon materials offer the necessary hydrophobicity for maintaining the separation of gas and liquid compartment and are adaptable to different electrode shapes. Thus, current densities of several hundred mA cm^−2^ are commonly reached with carbon-based GDEs (Hoang et al., 2017; Lv et al., 2018a; Ma et al., 2016b; Martić et al., 2019; Yang et al., 2020b). Commercially available, carbon-based GDLs are composed of a porous carbon tissue, for CO_2_ electrolysis mostly paper (nonwoven) or cloth (woven), and a hydrophobic microporous layer made of PTFE-bound carbon particles. Often, the carbon tissue itself also contains a certain amount of PTFE for improved hydrophobicity. This PTFE content, as well as the PTFE content of the MPL and the thickness of the tissue, markedly influence the performance of the GDL because of changes in hydrophobicity, resistance, and gas permeability. Kenis and colleagues found an optimum composition with 20 wt % PTFE in the MPL and 10 wt % in a 190 μm thick carbon tissue. A lower PTFE content in the MPL caused detachment of the carbon particles and thus low durability, whereas at higher PTFE contents, both in the MPL and in the tissue itself, the increased electrical resistance caused performance drops. Generally, thinner GDL tissues ensured improved gas diffusion, but tissues thinner than 190 μm did not provide a sufficient barrier between liquid electrolyte and gas compartment and showed extensive flooding (Kim et al., 2016).

Although carbon-based GDLs are used as state-of-the-art technology for CO_2_ electrolysis, their low mechanical, chemical robustness, and high price limit their upscaling to industrial relevance. Furthermore, the loss of hydrophobicity of carbon-based GDLs during electrolysis, as shown by Sargent and colleagues independently of the catalyst layer, frequently leads to blocking of CO_2_ diffusion pathways by flooding (B. Dinh et al., 2018a).

A frequently used modification of the GDE setup is the replacement of the carbon-based GDL by a PTFE substrate. An advantage of PTFE for the application as a GDL is its hydrophobicity, which reliably counteracts the penetration of electrolyte through the GDE and thus the flooding of the gas compartment. However, as PTFE is electrically insulating, the functionalities of gas diffusion and current distribution are decoupled in PTFE-based GDEs. Thereby, electrical contacting is realized via the catalyst layer, often leading to a less uniform distribution of electricity across the layer (G. Dinh et al., 2018b). Nevertheless, the successful application of those PTFE-based GDEs, achieving high current densities and selectivities for the target product, was reported in multiple studies. Using their 3D catalyst ionomer bulk heterojunction (CIBH) electrodes, Sargent and coworkers primarily obtained C_2+_ products at current densities of above 1 A cm^−2^, of which a large proportion was C_2_H_4_ with an FE of 65–75%. Although the GDL of the CIBH electrodes was based on PTFE, the CL was composed by Cu and a perfluorosulfonic acid (PFSA) ionomer (García de Arquer et al., 2020). As early as 2018, the Sargent group was using PTFE substrates to which firstly a Cu catalyst was applied, followed by layers of carbon nanoparticles and graphite. Afterwards, carbon black was utilized as a current collector via spray coating. Graphite and carbon NPs served to stabilize the catalyst layer. Stabilities of 150 h were achieved, with an FE for ethylene of 70% (B. Dinh et al., 2018a). Using a bimetallic Ag/Cu catalyst on a PTFE substrate in a flow cell with an AEM and 1 M KOH, they realized an FE of 41% for ethanol at 250 mA cm^−2^ and -0.67 V vs. RHE (Li et al., 2019). To give an overview about the state-of-the-art, Table 2 summarizes the properties of common commercially available GDLs.

**Table 2:** Properties of some selected commercially available GDLs according to manufacturer information

Manufacturer/Series	Type	Hydrophobic treatment	MPL	Thickness [μm]	TP resistance [mΩ cm^−2^]	IP resistance/conductivity	TP air permeability
Freudenberg (B. Dinh et al., 2018a) H series (Yang et al., 2020a)	Carbon paper	Some	Yes	141–230^a^	5–10	*R* = 0.6–1.1 Ω	0.6–90 s^b^ IP: 1.0–3.1 μm^2^
Sigracet BC series (Chen et al., 2020c; Kim et al., 2016)	Carbon paper	Yes (5 wt % PTFE)	Yes (23 wt % PTFE)	235–325	7.5–12	*σ* = 145–225 S cm^−1^	0.2–1.5 cm^3^cm^−2^ s^−1^^c^ 5–15 · 10^−12^ m^2^ IP: 1.4–2.7 μm^2^
AvCarb (Xing et al., 2021)	Carbon paper	Some (PTFE)	N/A	184–270	<14.5	N/A	3.5–18 s (100 cm^-3^)^d^
FuelCellsEtc ELAT (formerly E-TEK) (Chang et al., 2009)	Carbon cloth	Some	Some	406–490	0.17–0.34 (@ 181.4 kg load)	N/A	2.156 s^b^ 0.104 L (m^−2^ s^−1^ Pa^−1^)
QuinTech (Junge Puring et al., 2021)	Carbon cloth	Some	Some	360–410	<5 to <13	N/A	<10 - < 55^d^
Toray TGP-H (Aeshala et al., 2012; Albo and Irabien, 2016; Kim et al., 2016)	Carbon paper	N/A	N/A	110–370	*ρ* = 80 mΩ cm	*R* = 4.7–5.8 mΩ cm^−2^	1,500–2,500 mL mm (cm^−2^ h^−1^ mmAq^−1^)
Sterlitech Aspire (B. Dinh et al., 2018a)	ePTFE	No	No	76–305	Insulating	Insulating	0.07–8.0 ft^3^ (min^−1^ ft^−2^) @ 125 Pa

TP: through-plane, IP: in-plane.

a @ 1 MPa.

b according to ISO5636-5.

c according to ISO9237.

d according to Gurley.

### Ionomers

To enhance the mechanical stability of a GDE and improve its performance in CO_2_RR by creating a more hydrophilic surface, the electrode can be coated with an ionomer to produce a membrane-coated electrode (MCE). To avoid material loss from their GDE and tune its performance, Casada-Coterillo and colleagues tested different MCE setups for the production of methanol. Although some modifications led to an increase of the resistance of the electrode and had adverse effects on the methanol FE, coating with a mixture of chitosan and PVA with Cu-exchanged zeolite Y as a filler resulted in a marked increase from 40.1% to 68.0% compared to the uncoated variant (Marcos-Madrazo et al., 2019). Alternatively, ionomers can directly be added to and applied with the catalyst ink. Thereby, imidazolium has been shown to form a dense and positively charged layer around Ag-catalysts to repel protons. However, methylimidazolium-based polymers rapidly degrade in alkaline solution. On the example of CO formation it was therefore shown that ionomer-containing GDEs can be operated at higher current densities but with reduced long-term stability compared to ionomer-free electrodes (1,000 h at 200 mA cm^−2^ compared to 4,380 h at 50 mA cm^−2^ before flooding of the electrode) (Kutz et al., 2017). It is required to be aware of the fact that excessive amounts of ionomer can cause the adverse effect of blocking CO_2_ diffusion to the catalyst. Using polystyrene methyl methylimidazolium chloride (PSMIM) and polystyrene tetramethyl methylimidazolium chloride (PSTMIM) directly mixed into the catalyst ink, Masel and coworkers found that despite maximizing the catalyst surface area, ionomer contents above 8 wt % blocked CO_2_ diffusion (Kutz et al., 2017; Liu et al., 2018). A variation in the concentration of PSTMIM showed hardly any influence on the onset potential for CO_2_ reduction, but at 1 to 4 wt %, the obtained current density could be doubled (Kutz et al., 2017).

In addition to its amount, also the type of the applied ionomer is crucial for the performance of a GDE regarding reachable current densities and selectivity of the desired product. For instance, the use of the anion exchange polymer Fumasep FAA 30 was found to lead to higher current densities (200 mA cm^−2^ at 3.3 V cell voltage) in CO production with high selectivity, whereas the cation exchange polymer Nafion XL100 led to significantly lower current densities with lower FE for CO and increased HER (Pătru et al., 2019). The use of Sustainion XA7 (PMIM-Cl) in Ag porous carbon GDEs could even lead to three times higher current densities of up to 300 mA cm^−2^ for CO formation (Liu et al., 2018). In the case of formate as target product, the comparison of Nafion and a perfluorinated anion exchange (PFAE) polymer showed a low FE <5% for Nafion with simultaneously pronounced HER, while using PFAE, resulted in about an order of magnitude higher FE. In addition, the local pH value at the electrode was raised when using the PFAE ionomer, which results in a suppression of HER (Chen et al., 2020c).

Eventually, also the mode of application of an ionomer can influence its performance. In their CIBH electrodes, Sargent and colleagues controlled the orientation of their PFSA ionomer using a polar solvent for coating of the Cu catalyst in a way that the –SO_3_^-^ groups faced out toward the metal and the electrolyte. The goal was to create a layered PFSA structure in which ion and water transport are enabled by the hydrophilic –SO_3_^-^ domains and the gas transport by hydrophobic –CF_2_ domains, respectively. Instead of a partial current density of 64 mA cm^−2^ for C_2_H_4_ at the bare Cu electrode, up to 340 mA cm^−2^ were achieved by using the ionomer (García de Arquer et al., 2020).

### Variation of the operating parameters

Analogously to the H-type cells, the reaction parameters also influence the performance of CO_2_RR in flow cells. To gain insight into the complex mechanistic processes taking place in a GDE, Friedrich and coworkers investigated the influence of current density, temperature, and CO_2_ partial pressure on the CO_2_RR via electrochemical impedance spectroscopy (EIS). They used a carbon-based GDE with a tin catalyst and identified the charge-transfer reaction step by the respective resistance, which was decreasing with increasing current density or temperature. Furthermore, a decrease of CO_2_ partial pressure led to an increase in charge-transfer resistance. The authors analyzed two further processes via EIS taking place at the GDE during CO_2_RR: The conversion of CO_2_ with OH^−^ to form bicarbonate, and the liquid phase diffusion of CO_2_. Thereby, it was found that the resistance attributed to liquid-phase diffusion increases with increasing temperature, with the loss in CO_2_ solubility exceeding the enhanced diffusion at higher temperatures. The resistance of bicarbonate formation increases with higher current density, higher temperature, and lower CO_2_ partial pressure (Bienen et al., 2020).

When investigating the influence of the temperature on the Sn catalyst-based CO_2_RR to formate in a liquid-phase flow cell, Klemm and coworkers identified 50°C as the optimum operating temperature of the system regarding maximized current density and a formate FE of >80%. Higher and lower temperatures led to increased HER at the given current density of 1,000 mA cm^−2^. This can be assigned to the oppositional effects of reduced CO_2_ solubility and increased diffusion coefficients and reaction kinetics with increased temperature (Löwe et al., 2019). In addition, Lister and colleagues reported a reduction of the cell voltage by 1.57 V at 70 mA cm^−2^ while increasing the temperature from room temperature to 70°C when conducting the CO_2_RR to syngas with an Ag-based GDE in a liquid-phase electrolyzer (Dufek et al., 2011). Furthermore, in another study, they investigated the influence of both elevated temperature and pressure on the CO_2_RR using a Ag GDE at 225 mA cm^−2^. Increasing the pressure from atmospheric conditions to 18.7 bar led to a significant reduction of the overall cell voltage from 4.01 V to 3.71 V at 60°C. At 90°C, a cell voltage below 3 V with a CO FE of 82% could be achieved. This decrease in the overvoltage was confirmed by Sinton and coworkers, who utilized an Ag-based GDE cathode at pressures from 1.0 bar to 7.1 bar under alkaline conditions with KOH electrolyte. With 7 M KOH and a pressure of 7.1 bar, the system showed the highest half-cell energy efficiency of 81.5% compared to lower pressures, with a low cathodic overpotential for the reduction of CO_2_ to CO of 300 mV at 300 mA cm^−2^ as well as an FE of almost 100%. Furthermore, they found that the pressure influenced the selectivity of the reaction, whereby higher pressure led to high FEs for CO and lower pressures favored formate production (Gabardo et al., 2018). Contrasting observations were made by Schmid and colleagues when comparing the electrolysis of CO_2_ to CO at 5 and 25 bar. FEs for CO of >90% at current densities of up to 300 mA cm^−2^ were reached independently from the pressure. At 25 bar, higher cell voltages and thus lower energy efficiency were obtained. The authors concluded that elevated pressure operation offers no benefit for liquid-phase flow cells (Haas et al., 2018).

### Special designs for liquid-phase flow cells

Several attempts to adapt liquid-phase flow cells to special applications have been reported. For instance, an approach for the CO_2_RR to C_2+_ products is the coupling of electrolyzers in cascade setups. Using CO instead of CO_2_ electrolysis has been shown to be more suitable for reduction to higher alcohols, like ethanol and *n-*propanol, than CO_2_, because of the elimination of formate as a side product (Wang et al., 2018b). In this context, Hinrichsen and coworkers compared the reduction of both educt gases on Cu nanoparticles at 300 mA cm^−2^, showing 3-fold higher selectivity for ethanol and *n-*propanol for CO over CO_2_. As CO can be produced electrochemically by CO_2_RR with high FEs, the coupling of two electrolyzers in a two-step setup was proposed (Romero Cuellar et al., 2019, 2020). In a cascade setup, CO_2_ was reduced to CO in an Ag GDE flow cell, unconverted CO_2_ was subsequently removed from the product gas stream via absorption in NaOH, and the resulting CO gas was fed to the second flow cell. Two-step electrochemical reduction with a current density of 270 mA cm^−2^ for the first electrolyzer and 200 mA cm^−2^ at −0.82 V vs. RHE for the second electrolyzer, using Cu nanoparticle based GDEs, could yield an ethanol FE of 18% and an *n-*propanol FE of 7.5% (Romero Cuellar et al., 2020).

An alternative cell design, the so-called microfluidic flow cell, was established by Kenis and colleagues based on similar fuel cell architectures. Hereby, the cathode GDE and anode are not separated by a membrane but just by a channel of up to 1 mm, through which the electrolyte is passed. The laminar electrolyte flow thereby prevents the crossover of molecules (Jayashree et al., 2010; Lu et al., 2017; Monroe et al., 2017; Whipple et al., 2010). Possible advantages of omitting the use of a membrane are reduced capital cost as well as lower ohmic losses affecting the energy efficiency (Esposito, 2017; Kibria et al., 2019; Lu et al., 2017). However, circulation of the electrolyte is not possible when liquid products are formed and upscaling of the electrolyzer design is complex (Kibria et al., 2019).

### Limitations of liquid-phase flow cells & gas diffusion electrodes

Besides the advantages of liquid-phase flow cell setups equipped with a GDE mentioned at the beginning, a general problem of the use of aqueous electrolytes is the pronounced formation of hydrogen. Because CO_2_RR takes place at potentials close to HER, both reactions are competing (Garg et al., 2020). Furthermore, the dilution of liquid products in the electrolyte increases the cost for product separation (Gabardo et al., 2019).

Another frequently documented problem when using liquid electrolytes is flooding of GDEs, *i*.*e*. penetration of the electrolyte into the electrode structure, which is accompanied by massive performance losses (Larrazábal et al., 2019; Malkhandi and Yeo, 2019). During flooding, the CO_2_ diffusion paths of the CL are blocked and higher current densities can no longer be obtained (B. Dinh et al., 2018a; Gabardo et al., 2019; Leonard et al., 2020). The decreased availability of CO_2_ leads to preferential HER in the whole area (Leonard et al., 2020). There are various causes for the phenomenon of electrode flooding, e.g. macroscopic pressure imbalances, the development of surface wettability, and evaporation/condensation effects (Leonard et al., 2020). For carbon-based GDEs, hydrophobicity losses during operation are assumed to be the decisive factor (G. Dinh et al., 2018b; Gabardo et al., 2019). Investigations on the flooding behavior of GDEs with silver showed that GDEs exposed to higher current densities retain less of their original hydrophobicity, *i*.*e*. there is a correlation between the current density and the flooding of the GDEs (Leonard et al., 2020). Moreover, the GDEs showed a contrary behavior regarding the amount of CO in the product gas flow and the capacitance of the cathode. Based on the observation that the capacitance increased with a delay of about 15 min after the drop in CO mole fraction in the product gas, it is assumed that the penetration of the electrolyte solution is not the sole cause of the performance loss. According to Rufford and coworkers, crystallites on the back of the flooded GDEs and at the inlet of the cathode flow field indicate a connection between carbonization and flooding. The corresponding hypothesis to this observation is that the electrode failure is caused by the precipitation of carbonates, which is followed by rapid flooding and inhibits further CO_2_ flow (Garg et al., 2020). When studying the flooding behavior of their GDEs, Brushett and coworkers noted that the collapse of their GDEs always happened after the passage of a certain cumulative charge. They assumed the existence of a material-specific carbonate threshold value, whose attainment inevitably leads to electrode failure (Leonard et al., 2020). Furthermore, the formation of carbonate and bicarbonate salts because of the reaction of CO_2_ with alkaline electrolytes can change the electrolyte pH and conductivity and thus affect the GDE performance (Gabardo et al., 2019; Kibria et al., 2019). Burdyny and coworkers took a closer look into the role of carbon-based GDLs in GDE flooding and found out that in KHCO_3_, HER activity of the GDL itself can be linked to flooding, making it largely independent from the actual CO_2_RR. A more active catalyst with decreased onset potentials for CO_2_RR is assumed to prevent HER at the GDL and the accompanying reduction of capillary pressure initiates flooding (Yang et al., 2020a).

To circumvent flooding, PTFE-based membranes are increasingly used as GDLs. Swiegers and colleagues showed that flooding of a PTFE GDL was just observed at an overpressure of 5.8 bar on the liquid side, which is a magnitude higher than for conventional, carbon-based GDLs (Shafaque et al., 2020). In addition, Sargent and coworkers used PTFE substrates as GDL, with a carbon black layer spray-coated onto the catalyst as current collector (G. Dinh et al., 2018b; Gabardo et al., 2019; Wang et al., 2020b). Thereby, as already mentioned above in this chapter, gas-diffusion functionality and current distribution are decoupled. A drawback of the PTFE-based electrodes is the lack of through-plane conductivity. The insulating PTFE GDL complicates the implementation of such GDEs into electrolyzer stacks. An approach to facilitate carbonate removal at the gas-liquid interface of the GDE and avoid wetting is the integration of application-specific microstructures (Leonard et al., 2020).

In addition to salt precipitation and flooding of the GDE, an additional problem is the potential product crossover through the GDE (Ma et al., 2020b). It has been shown that a considerable amount of alcohols, such as *n*-PrOH and EtOH, but especially acetaldehyde, evaporate through the GDE. In cases where only the liquid phase is analyzed after electrolysis, the FEs of these products can be significantly underestimated. The formation of bubbles of gaseous products implies another challenge, as the catalyst layer can be damaged or destroyed (Malkhandi and Yeo, 2019).

Overall, liquid-phase electrolyzers represent a promising cell architecture that has been proven to be capable of reducing CO_2_ to C_2+_ products at high current densities. However, up to now, there are hardly any studies dealing with the development of gas diffusion electrodes, which are scalable and enabling current densities relevant for industrial scale simultaneously with high FEs (Chen et al., 2020c). Yet, further optimization regarding long-term stability of the GDE and the reduction of the cell voltage is required for industrial implementation. Ohmic losses can occur with poorly conductive liquid electrolytes (Larrazábal et al., 2019). Membrane electrode assemblies (MEAs), which eliminate the need for a liquid electrolyte, are one approach for improved GDE operation and will be discussed in chapter 5.

### H-type cells & liquid-phase flow cells in direct comparison

In summary, the use of gas diffusion electrodes within a flow cell has several advantages over the application of H-type cells. A change from the planar cathode geometry of an H-cell to a GDE leads to an overall improvement of the current density in the range of several orders of magnitude because of an increased electrochemically active surface, as well as an improved mass transfer (Weng et al., 2019). It is possible to reduce diffusion limitations, because more CO_2_ reaches the catalyst surface and products are discharged (Salvatore et al., 2018). As a result, the yield of CO_2_RR products in flow cells is up to two orders of magnitude higher than in H-type cells (Malkhandi and Yeo, 2019). Furthermore, switching from an H-type to a flow cell can influence the selectivity, as shown in the example of a Cu-ZnO catalyst. Contrarily to the H-type cell, ethanol formation was favored over ethylene formation in the flow system (Ren et al., 2019).

Table 3 gives an overview of recent developments (2018–2021) in the reduction of CO_2_ with a focus on the production of multicarbon alcohols, including the cells used and ion exchange membranes. It is difficult to make a direct comparison of the influence of individual parameters on FEs for alcohol production, as each publication shows many differences concerning the process design as well as the operating parameters. However, it becomes visible that the highest current densities are clearly obtained using flow cell architecture. The benchmark for industrially relevant partial current densities and overall cell voltages is at least 300 mA cm^−2^ at a maximum of 2.0 V, which was not demonstrated in an H-type cell yet (Jouny et al., 2018; Kibria et al., 2019). Regarding CO_2_RR to multicarbon alcohols, selectivity poses a second major challenge. Product yields marked with “C_2+_^”^ often contain high amounts of C_2_H_4_, which is in many cases the reason for the higher FEs obtained. In copper-based electrodes, ethylene is generally formed preferentially to ethanol (Ren et al., 2016). Nevertheless, some publications describe FEs for multicarbon alcohols of 30% and higher. Best results were obtained with 41% FE_EtOH_ at 124 mA cm^−2^ for a catalyst consisting of FeTTP[Cl] (TPP = tetraphenylporphyrin) on Cu and sputtered onto a PTFE substrate and with 41% FE_EtOH_ at 250 mA cm^−2^ for an Ag_0.14_/Cu_0.86_ catalyst (Li et al., 2019, 2020). The other catalysts summarized in Table 3 achieving higher yields generally reached current densities below 20 mA cm^−2^. The Ag-graphene nitrogen-doped carbon foam (NCF) catalyst reached a FE_EtOH_ of up to 85%, but the current density was less than 1 mA cm^−2^ (Lv et al., 2018b).

**Table 3:** Overview of recent developments in the reduction of CO_2_ to multicarbon alcohols between 2018 and 2021

FE	Current densities [mA cm^−2^]	Potential vs RHE [V]	Catalyst	Electrolyte	Membrane	Ref
**H-type cells**						
52.3% EtOH	<15 total	−0.3	Au@Cu_2_O yolkshell NPs on carbon cloth	0.1 M KHCO_3_	PEM (Nafion 117)	(Zhang et al., 2020a)
80% C_2_ products	21C_2_ products	−1.09	Reconstructed porous Cu	0.1 M KHCO_3_	PEM (Nafion 117)	(Han et al., 2020b)
33.7% EtOH 6.9% PrOH	8.67 EtOH 1.8 PrOH	−1 −0.9	Ag_15_Cu_85_	0.5 M KHCO_3_	–	(Dutta et al., 2020)
64.6% EtOH 8.7% PrOH	ca. 8 EtOH ca. 1.2 PrOH	−1.05	Cu NPC	0.2 M KHCO_3_	PEM (Nafion 212)	(Han et al., 2020a)
≈80% C_2+_ products	ca. 8C_2+_ products	−0.9	CuO_x_	0.1 M CsHCO_3_	AEM (Selemion AMV)	(Jeong et al., 2020)
ca. 70% C_2+_ products	40 to 50C_2+_ products	−1.05	Cu oxide-/hydroxide-derived	0.1 M KHCO_3_	PEM (Nafion 117)	(Lei et al., 2020)
78% EtOH	ca. 0.2 EtOH	−0.56	Micropores in N-doped mesoporous carbon	0.1 M KHCO_3_	PEM	(Song et al., 2020)
16.4% EtOH 14.9% C_2_H_4_	4.1 EtOH	−1.1	Cu-OD + Ag (20 nm)	0.1 M KHCO_3_	AEM (Selemion AMV)	(Ting et al., 2020)
80% C_2+_ products with 40% C_2_H_4_, (EtOH, PrOH)	ca. 4 total	−1.2	Cu NPs + poly-aniline	0.1 M KHCO_3_	AEM (QAPPT)	(Wei et al., 2020)
48% EtOH	2.5 total	−0.8	Cobalt corrole complex on carbon paper	0.1 M NaClO_4_	–	(Gonglach et al., 2019)
25% C_2_H_4_ 5% EtOH	–	−1.8	Cu(OH)_2_/Cu	0.1 M NaHCO_3_	PEM (Nafion)	(Iijima et al., 2019)
21% C_2_H_4_ 29% EtOH	18 total	−1.0	Cu@Cu_2_O	0.1 M KHCO_3_	–	(Shang et al., 2019)
69% C_2+_ products	45.5C_2+_ products	−1.0	CuO_x_	0.1 M CsHCO_3_ +0.1 M CsI	AEM (Selemion AMV)	(Gao et al., 2018)
60% C_2+_ products (32% C_2_H_4_)	68 total 40C_2+_ products	−0.96	Cu-NCs	0.25 M KHCO_3_	PEM (Nafion 117)	(Jiang et al., 2018b)
79.1–85.2% EtOH	0.31 total	−0.5 to −0.7	Ag-graphene-NCF	0.1 M KHCO_3_	AEM	(Lv et al., 2018b)
79% C_2_ products (52% C_2_H_4_ 27% EtOH)	10C_2_ products	−1.1	Boron-doped Cu	0.1 M KHCO_3_	PEM (Nafion 117)	(Zhou et al., 2018)
40.3% C_2_H_4_ 16.7% EtOH 12.1% *n*-PrOH 4.4% AcOH	20 to 30 total	−1.3	CuPb-0.7/C	0.1 M KHCO_3_	PEM (Nafion 117)	(Wang et al., 2020a)
72% C_2+_ products (35.9% EtOH)	8.75 EtOH	−1.25	Dodecanethiol-modified CuBr	0.5 M KCl	PEM (Nafion 117)	(Wang et al., 2021)
13.7% *n*-PrOH	1.15 PrOH	−0.65	PdCu alloy foam (Pd_9_Cu_91_)	0.5 M KHCO_3_	PEM (Nafion 117)	(Rahaman et al., 2020)
20.2% EtOH 2.1% PrOH 33.6% C_2_H_4_	–	−1.1	OD-Cu_90_Zn_10_ cubes	0.1 M KHCO_3_	AEM (AHO, AGC Inc.)	(da Silva et al., 2020)
32% EtOH	<10 total	−1.1	Multimetallic CuAgHg	0.1 M KHCO_3_	–	(Kim et al., 2020b)
53% EtOH 18% *n*-PrOH	30 to 35 total	−1.08	Defect-site rich Cu	0.1 M KHCO_3_	–	(Gu et al., 2021)

**Flow cells**						
78% C_2+_ products (C_2_H_4_ 49%)	466C_2+_ products	−0.73	ZnO-layer on top of Cu on carbon paper	1 M KOH	–	(Zhang et al., 2020c)
85.5% C_2-4_ (15% EtOH; 65.2% C_2_H_4_)	800 total	−0.89	Fluorine-modified Cu	1 M KOH	AEM	(Ma et al., 2020c)
52% EtOH	156 EtOH	−0.68	Cu + N-C on PTFE substrate	1 M KOH	AEM	(Wang et al., 2020b)
41% EtOH	124 EtOH	−0.82	FeTTP[Cl] on Cu, sputtered on PTFE substrate	1 M KHCO_3_	AEM (Fumapem FEE-3-PK-130)	(Li et al., 2020)
75.2% C_2+_ products	267C_2+_ products	−0.61	Multi-hollow Cu oxide	2 M KOH	AEM	(Yang et al., 2020b)
36.9% alcohols 8.6% MeOH 28.3% EtOH	20 total	−0.67	Cu + Bibased metal-organic frameworks (MOFs)	0.5 M KHCO_3_	PEM (Nafion 117)	(Albo et al., 2019)
55% EtOH	16.2 total	−1.2	Cu-N-C	0.1 M CsHCO_3_	AEM (Selemion AMV)	(Karapinar et al., 2019)
41% EtOH	250 total	−0.67	Ag_0.14_/Cu_0.86_	1 M KOH	AEM (Fumasep FAA-3-PK-130)	(Li et al., 2019)
61.7% C_2+_ products 42% C_2_H_4_ 14% EtOH 5% PrOH	185C_2+_ products	−0.59	Cu-Cu_4_O_3_	2.5 M KOH	PEM (Nafion 117)	(Martić et al., 2019)
48.6% C_2+_ products	97C_2+_ products	−0.68	ZnO/CuO	1 M KOH	AEM	(Ren et al., 2019)
40% C_2_ products (C_2_H_4_, EtOH)	234 total	−1.17	Cu_x_O	2 M KOH	PEM	(Xiang et al., 2019)
60% C_2_H_4_ 25% EtOH	180C_2_H_4_	−0.7	Cu-Ag	1 M KOH	AEM (Fumatech FAP-375-PP)	(Hoang et al., 2018)
84% C_2+_ products (>60% C_2_H_4_)	336C_2+_ products	−0.68	CuCl-derived Cu	3 M KOH	AEM	(Kibria et al., 2018)
62% C_2+_ products (C_2_H_4_, EtOH PrOH)	411C_2+_ products	−0.67	Cu-NPs	1 M KOH	AEM (FAA-3 Fumatech)	(Lv et al., 2018a)
32% C_2+_-alcohols (25% EtOH 7% PrOH)	120C_2+_ alcohols	−0.92	Cu_2_S-Cu-V	1 M KOH	AEM	(Zhuang et al., 2018)
29.9% EtOH 1.43% n-PrOH 16.3% AcOH	400 total	Min. −1.5	CuPb-0.7/C	1 M KOH	AEM (Fumasep FAB-PK-130)	(Wang, P. et al., 2020)
64% C_2+_ products (15% EtOH)	210 total	−0.7 to −0.75	P-doped Cu (Cu_0.92_P_0.08_)	1 M KOH	PEM (Nafion 115)	(Kong et al., 2021)
52.4% C_2+_ alcohols	282.1 total	−0.9	N-doped graphene quantum dots on Cu-OD Cu-nanorods	1 M KOH	AEM (Fumasep FAA-3-PK-130)	(Chen et al., 2020a)
<30% EtOH	400 total	–	Ag_2_Cu_2_O_3_	1 M CsHCO_3_	AEM (Fumasep FAB-PK-130)	(Martić et al., 2020)
40% C_2_H_4_ 20% EtOH	–	−0.5	Cu electrodeposited on carbon paper	1 M KOH	AEM (Fumatech)	(Hoang et al., 2017)
52% EtOH 15% *n*-PrOH	100 total	−0.95	Defect-site rich Cu	1 M KOH	AEM	(Gu et al., 2021)

With regard to the membranes used, Table 3 shows that AEMs were used more frequently than PEMs, especially in flow cell architecture. The most frequently used electrolytes are KHCO_3_, CsHCO_3_, and KOH. It is required to be aware of the fact that neither the influence of the membranes nor the electrolytes a trend can be derived based on the data given in the table, as completely different catalysts were used.

## Solid-electrolyte cells

**Figure 8:**
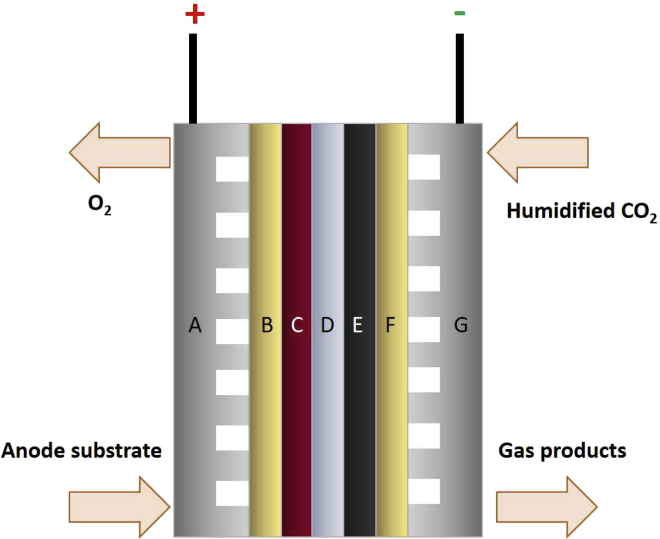
Zero gap-type electrolyzer (A–F) Schematic drawing. (A) anode flow field; (B) anode porous transport layer (PTL), commonly Ti mesh; (C) anode catalyst layer (CL), commonly IrOx; (D) ion-exchange membrane (IEM); (E) cathode CL; (F) cathode PTL, commonly carbon-based or PTFE-based; (G) cathode flow field. As anode substrates, deionized water, aqueous KOH or KHCO_3_ or humidified N_2_ can be used.

In a membrane electrode assembly (MEA), also called zero-gap arrangement, both the cathodic GDE and the anode are in direct contact with the ion exchange membrane, which functions as electrolyte (Gabardo et al., 2019; Larrazábal et al., 2019). The substrates are led to the electrodes from the reverse side, as schematically shown in Figure 8.

The problem of mass transfer limitation of CO_2_ through the aqueous electrolyte, as it occurs in H-type cells, is commonly eliminated in MEA setups, which guarantee high CO_2_ concentrations at the catalyst, as well as lower ohmic losses because of the renunciation of the liquid electrolyte between cathode and anode. This omission also prevents the flooding of the GDE as well as CO_2_ consumption by the electrolyte and contamination that can affect the catalytic performance. In consequence, gas-phase electrolyzers show stable performance regarding cell voltage and product selectivities (Gabardo et al., 2019; Kibria et al., 2019; Lin et al., 2020). A drawback is the possible blocking of the pores of the GDE with liquid products or water at high current densities, impairing the availability of CO_2_ at the catalyst. For this reason, the removal of liquid products from the GDE is of great importance (Kibria et al., 2019). The migration of liquid products to the anode site and subsequent oxidation could decrease the FEs of those products.

Water management plays a crucial role in the design of MEAs. The water necessary for CO_2_RR is usually provided via a humidified CO_2_ stream, which enables effective management of the reactants and the resulting conversion rates (Blommaert et al., 2019; Lee et al., 2015; Wang et al., 2018a). The most common operation mode of a MEA is with a humidified CO_2_ stream to the cathode and a liquid anode substrate. Alternatively, the use of a dry CO_2_ stream in combination with a liquid anode substrate was reported, but accompanied by losses in ionic conductivity of the IEM (Shafaque et al., 2020). In addition, the use of humidified gas streams as both cathode and anode substrate resulted in membrane dehydration when the MEA was operated at room temperature. Increasing the temperature to 80 °C led to sufficient hydration of the membrane, but the FE for CO decreased markedly from around 80% at 25 °C to less than 20% at a cell voltage of 2.5 V (Weng et al., 2019). Process parameters like temperature and pressure generally influence the electrochemical performance of solid-phase electrolyzers. An increase in operation temperature of MEA setups was often reported to increase achievable current densities and FEs of the target products (Gabardo et al., 2019; Lee et al., 2018; Sebastián et al., 2017). However, it is required to remember the fact that membrane stability and water crossover limit the maximum temperature in a gas-phase electrolyzer (Kibria et al., 2019). Endrődi and coworkers performed electrolysis of moistened CO_2_ to CO in a zero-gap MEA stack at pressures up to 10 bar. They pointed out multiple factors that facilitate CO_2_ reduction at elevated pressure, *i*.*e*., thermodynamically favored CO_2_RR because of the higher CO_2_ activity and lower relative water amounts, enhanced mass transport to the GDE, and improved mechanical contact with the AEM. A partial current density for CO of 300 mA cm^−2^ was achieved with a CO/H_2_ ratio >20 at 3 V cell potential. By flushing the cell with deionized water every hour, the performance was maintained for 8 h (Endrődi et al., 2019). However, the detailed influence of the process parameters on the performance of a MEA is not completely understood yet. To enable more directed research on MEA systems, Sinton and coworkers used numerical modeling to assess the influence of potential, CO_2_ partial pressure and AEM thickness, porosity, and charge on carbonate and liquid product crossover as well as CO_2_ utilization and energy efficiency. The model confirmed experimental observations on the positive influence of high current densities, high temperatures, and decreased partial CO_2_ pressure on EtOH selectivity. With this detailed investigation, they proved computer-based research as a powerful tool to gain insight into the processes taking place in a MEA (McCallum et al., 2021).

Notably, catalysts that were incapable of the formation of certain CO_2_RR products in the presence of aqueous electrolytes could be reconsidered for the application in a MEA. Perathoner and colleagues showed that Fe, a metal that is not capable of reducing CO_2_ to higher alcohols and hydrocarbons under conventional liquid-phase electrolysis conditions, can be active toward the formation of these products under liquid electrolyte-free conditions in a solid-phase electrochemical cell. They assign the different reaction behavior to the higher CO_2_ coverage of the catalyst surface (Genovese et al., 2013).

### Influence of the membrane type

Compared to liquid-phase electrolyzers, the influence of the membrane is more pronounced in a solid-phase electrolyzer because of the close contact and the omission of the liquid electrolyte, which could for example buffer pH effects. Similar to the other electrolyzer setups, PEMs, AEMs or BPMs can be used (Kibria et al., 2019; Lin et al., 2020). A challenge linked to PEM-based systems is the formation of an acidic environment at the GDE, possibly resulting in increased HER rates at high current densities or prolonged reaction times, making control of the local conditions at the cathode highly important (Dewulf and Bard, 1988; Kibria et al., 2019). Newman and colleagues described the use of an aqueous KHCO_3_ pH-buffer layer between catalyst and membrane to prevent the acidification (Delacourt et al., 2008). The proton availability at the cathode is lower in AEM-based MEAs. Different membrane types can thus affect the product distribution (Kibria et al., 2019). A comparison of Nafion PEMs with Fumasep alkaline AEMs in a MEA using a Cu/carbon nanotube (CNT) catalyst showed a preferred CO_2_RR in the AEM setup (Wang et al., 2018a). Furthermore, by means of a comparison between PEMs made of Nafion and sulfonated poly(ether ether ketone) and an alkali-doped polyvinyl acetate AEM in a MEA setup, the influence of the membrane choice on the selectivity of CO_2_RR was pointed out. Although the PEM utilization rather yielded methanol and formaldehyde, the use of AEMs led to the formation of CO as well as a small amount of formic acid (Aeshala et al., 2012). However, carbonate crossover harshly limits the CO_2_ utilization when operating AEM-based systems, resulting in cost-intensive CO_2_ recovery steps. To overcome this drawback while still maintaining the necessary alkaline conditions at the cathode, Sinton and coworkers combined a Nafion 117 PEM with a PTFE-based and Cu-based electrode coated with a so-called permeable CO_2_ regeneration layer (PCRL) of the anion-exchange polymer Aemion AP1-CNN5-00-X. In this setup, they were able to achieve comparable performance like the AEM-based system (40% FE toward C_2_H_4_ and 55% FE toward C_2+_ products, respectively, at 100 mA cm^−2^ and 4.2 V cell voltage) with a CO_2_ conversion efficiency as high as 85%. Notably, the use of DI water as anode substrate is necessary in this system, because the migration of other cations than protons would result in carbonate precipitation at the PEM-PCRL interface (O’Brien et al., 2021).

Because of better control of the proton availability, MEA-based CO_2_ electrolyzers can be operated at exceptionally high current densities while maintaining high FEs for the target products. For ease of classification, benchmarking examples are given in Table 4. However, changes in selectivity caused by changes in the cathode environment, and in case of liquid anode substrates, water migration from the anode limit the applicable current densities also for MEA-based electrolyzers. At current densities above 200 mA cm^−2^, Seger and coworkers observed a selectivity change toward the formation of liquid products and carbonate, which they attributed to the locally enhanced pH value at the cathode. Furthermore, with increasing current densities, also HER became more pronounced, presumably because of flooding of the cathode by migrated anode substrate (Larrazábal et al., 2019).

**Table 4:** Benchmarking examples for performances of MEA-Based electrolyzers

MEA setup	Product (FE)	Current Density [mA cm^−2^]	Cell voltage [V]	Ref
WE: Ag membrane M: Sustainion AEM CE: IrO_x_/C	CO (≈80%)	250	3.3	(Larrazábal et al., 2019)
WE: Cu NPs/PTFE M: Sustainion AEM CE: IrO_x_/Ti	C_2+_ (≈80%) C_2_H_4_ (≈50%)	150	4	(Gabardo et al., 2019)
WE: CuTPI/PTFE M: Sustainion X37-50 AEM CE: Ti-IrO_x_ mesh	C_2_H_4_ (66%)	≈350	≈4.4	(Ozden et al., 2020)
WE: Ag NP/carbon paper M: PiperION AEM CE: IrO_x_/Ti	CO	1004 (partial)	3.4	(Endrődi et al., 2020)
WE: FeTPP[Cl]/Cu/PTFE M: Sustainion AEM CE: IrO_x_/Ti mesh	EtOH (41%)	≈500	3.7	(Li et al., 2020)
WE: Defect-site-rich Cu/Freudenberg H14C9 M: Sustainion AEM CE: IrO_2_/Ti mesh	EtOH + *n*-PrOH (60%)	200	3.5	(Gu et al., 2021)

CuTPI: copper/tetrahydro-phenanthrolinium/ionomer; M standing for membrane.

Overall, MEA-based electrolyzers already show promising results for CO_2_RR and constitute an important step in the direction of the industrialization of CO_2_ electrolysis. Numerous catalysts for CO_2_RR have already been designed and studied in detail on a laboratory scale, but regarding any future implementation in industrial scale electrolyzers, there is a lack of knowledge about their effective integration in MEAs and their operation (Weekes et al., 2018). Furthermore, many studies on MEAs have so far been performed at low current densities, which are not relevant for industrial applications (Gabardo et al., 2019).

### Solid-oxide electrolysis cells (SOECs)

**Figure 9:**
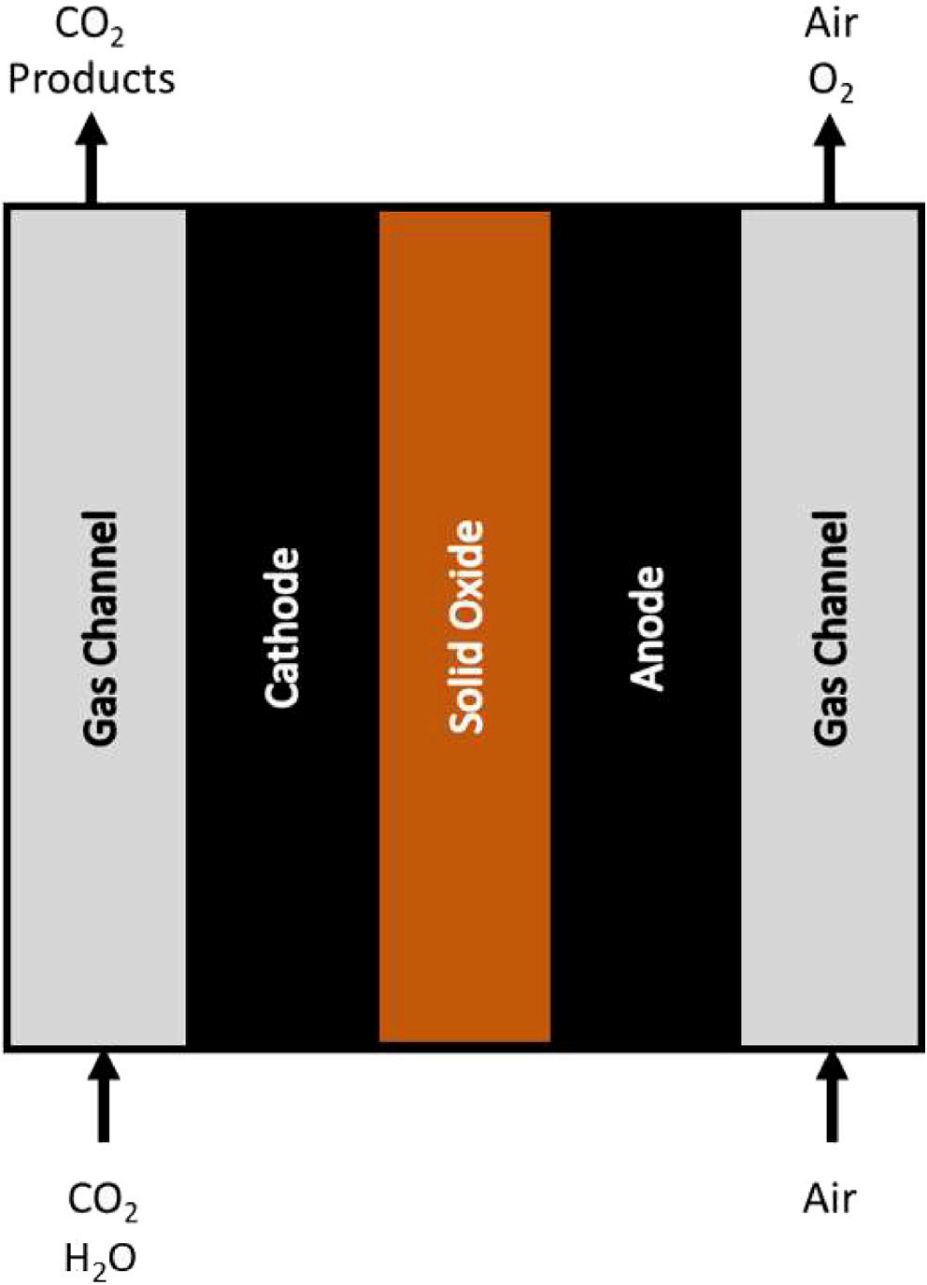
Assembly of a high temperature solid oxide-based electrolyzer

Solid-phase electrolyzers using ceramic electrolytes enable the direct production of syngas out of CO_2_ and water at high current densities because of the improved kinetics at elevated temperatures (Liang et al., 2020; Zhang et al., 2017). In this set-up, cathode and anode are separated by a layer of oxide and temperatures of >600°C are required for sufficient conductivity (Song et al., 2019). Common ceramic electrolytes are based on oxidic compounds of ceria, zirconia, and lanthanum gallate. Cathode materials usually deployed are porous metal/ceramic composites (cermets), for example yttria-stabilized-zirconia with Ni (Bidrawn et al., 2008; Ebbesen and Mogensen, 2009; Inaba, 1996; Ishihara et al., 2006; Yamamoto et al., 1998; Yue and Irvine, 2012; Zhang et al., 2017). The high-temperature solid electrolytes can be classified into two groups. In the case of oxygen ion (O^2−^) conductors, O^2−^ ions originate from the cathodic reaction of H_2_O, migrate from the cathode to the anode and are oxidized there. The CO_2_ molecules are reduced at the cathode with activated hydrogen. With proton-conducting oxides, H_2_O is oxidized to O_2_ at the anode and H^+^ migrates to the cathode, where it reacts with CO_2_ (Lin et al., 2020; Zhang et al., 2017). Figure 9 shows the scheme of a SOEC setup.

Despite the favorably high current densities they allow, the high temperatures that are required for the operation of SOECs lead to high energy demand and considerable technical complexity. Challenges are the sealing of the cell as well as metal particle oxidation, carbon deposition, and electrolyte degradation because of phase-changes at high temperatures (Liang et al., 2020; Zhang et al., 2017). Although the possible CO_2_ reduction products of solid-oxide electrolysis itself are rather limited, their subsequent conversion could offer a promising pathway to higher hydrocarbons (Liang et al., 2020). Sargent and coworkers followed this approach and coupled CO_2_ reduction to CO in a SOEC with subsequent conversion to ethylene in a MEA-based cascade approach. They reached around 48% reduction in energy input compared to a direct CO_2_ to ethylene route, because the energy-intensive reprocessing of the electrolyte and anode product gas stream caused by carbonate formation were completely avoided. Thereby, 95% FE for CO were reached in the SOEC at a current density of 815 mA cm^−2^ (Ozden et al., 2021).

## Product separation

After conversion, the diverse products of electrochemical CO_2_ reduction accumulate in rather complex mixtures. The separation of the single compounds from the gas stream and electrolyte requires further process steps to be considered for economic operation.

### Gas-phase products

**Figure 10:**
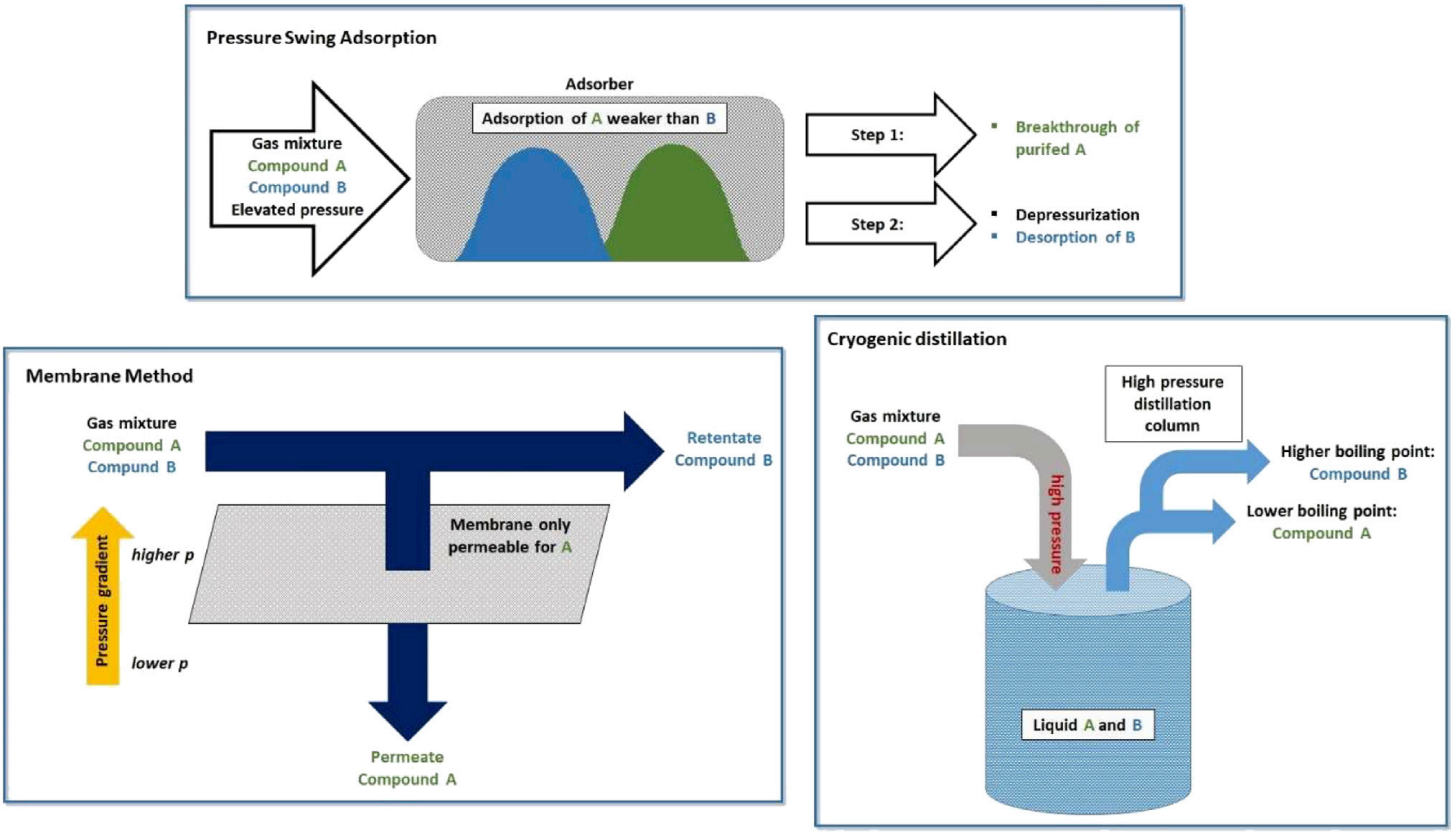
Gas separation processes Schematic drawings. Top: pressure swing adsorption (PSA). Bottom left: membrane method. Bottom right: Cryogenic distillation.

As CO_2_ reduction can yield various gas-phase products, they have to be separated from each other and the remaining CO_2_ stream for further processing. In Figure 10, different procedures for gas-separation are illustrated.

Greenblatt and colleagues provided a comprehensive overview of possible product separation steps for CO_2_ reduction products (Greenblatt et al., 2018). A possible method for gas separation is the pressure swing adsorption (PSA), which is also applied for industrial biogas applications (Bauer et al., 2013; Greenblatt et al., 2018; Ribeiro et al., 2011; Wiheeb et al., 2016; Zhu et al., 1991). This method is based on the adsorption of the desired product onto a porous material and its subsequent release under reduced pressure. The energy demand is based on the electricity needed for pressure control (Greenblatt et al., 2018; Wiheeb et al., 2016). Spurgeon and coworkers proposed the use of PSA to separate and recover unconverted CO_2_ from the gas stream. The technology could also be used for the regeneration of CO_2_ and the separation of CO in a two-step electrochemical reduction setup (Spurgeon and Kumar, 2018). Gas product separation via membranes also represents a viable option. Membranes used for this purpose are usually based on polymers which allow for the passage of the desired substances while preventing it for others (Greenblatt et al., 2018). This method promises low energy consumption, but challenges like the plastification of the membranes are still to be overcome (Sanders et al., 2013). Besides membrane separation, Greenblatt and colleagues proposed cryogenic distillation and pressurized liquefaction to separate a hypothetical mixture of CO_2_RR gas products. For cryogenic distillation, a mixture of gases with different condensability is liquefied via increased pressure. The different species can then be separated by distillation to their corresponding boiling point. Although the process is energy intensive, it can yield gas products in high purity. The authors described the possible liquefaction of ethylene and CO_2_ at elevated pressures of 50 and 70 bar, respectively. However, this process is not suitable to yield products with high purity when separation is done simply by raising the pressure again (Greenblatt et al., 2018).

### Liquid-phase products

Alcohols, organic acids or aldehydes represent attractive target products of the electrochemical CO_2_ reduction. Distillation is widely used in industry to separate liquid products, based on the control of temperature and pressure to evaporate the components selectively. The evaporation requires a high energy input, although the heat can partially be recovered (Greenblatt et al., 2018; Halvorsen and Skogestad, 2011; Kiss et al., 2012). However, the required energy is increased with more diluted target products, impeding their separation from the electrolyte stream (Greenblatt et al., 2018; Huang et al., 2008). To effectively increase the concentration of liquid products, the electrolyte can be recycled and passed multiple times through the electrode compartments. However, degradation effects connected to higher concentrations as well as an increased migration of CO_2_RR products to the anode compartment have to be taken into account (Jouny et al., 2018; Ma et al., 2017). The situation can furthermore be complicated by difficulties to separate the obtained products (Jouny et al., 2018). The azeotropic behavior of ethanol-water mixtures restricts the product purity obtained via distillation. The obtained purity can be optimized by the addition of another component like benzol or cyclohexane; however, these components have to be separated from the desired product again, resulting in additional process steps and separation costs (Greenblatt et al., 2018). Furthermore, the use of vacuum distillation reduces the required energy (Beebe et al., 1942; Greenblatt et al., 2018). The combined use of membrane technology and distillation, the so-called pervaporation, is another possibility to yield pure liquid products like ethanol. The desired product has to permeate the membrane selectively, where it is evaporated under reduced pressure and subsequently collected with a condenser (Al-Asheh et al., 2004; Greenblatt et al., 2018; Sanders et al., 2013). Some liquid products can also be extracted via liquid-liquid extraction. For example, the addition of solvents such as *n*-pentanol or higher alcohols allows for the preferential dissolution of *n*-propanol in the organic phase which is not miscible with the aqueous phase and thus can be separated (Greenblatt et al., 2018; Stoicescu et al., 2011). Because of their similar boiling points, also the separation of formic acid from water requires alternative methods. *BASF SE* described a liquid-liquid extraction process for formic acid from aqueous solutions with amides as potential extractants and subsequent distillation. In addition, the separation of formic acid, ethanol, and propanol can be facilitated by the addition of salts before extraction (Card and Farrell, 1982; Greenblatt et al., 2018; Li et al., 2000; Singh and Bell, 2016). Sing and Bell postulated the use of a concentrated Cs_2_CO_3_ electrolyte, leading to a phase separation of produced ethanol into microemulsion, which is subsequently collected in a liquid-liquid extractor, while the Cs_2_CO_3_ solution is recycled (Singh and Bell, 2016). For the use of additional solvents or salts, efficient regeneration of those additives is important to limit the cost of the separation steps (Greenblatt et al., 2018).

Altogether, the capture of CO_2_ as well as the separation of the desired products has a significant influence on the successful realization of industrial CO_2_ reduction. Optimization and efficient integration of those process steps can improve the overall efficiency and thus economic feasibility. With further technical improvement, the direct air capture (DAC) of CO_2_ could prospectively become an attractive CO_2_ source, facilitating the reduction of atmospheric CO_2_. As the separation of different products requires complex process steps, catalyst designs achieving high FE are of great importance. Furthermore, process optimization in direction of product streams that are less diluted with CO_2_ would be desirable.

## Techno-economic assessment

Although the electrochemical reduction of CO_2_ is a promising technology for both energy storage and carbon supply applications, the overall process has to be economically feasible for a successful industrial implementation. Techno-economic assessments have been issued to determine desired products as well as target parameters that the system has to meet. Thereby, every process step, ranging from CO_2_ capture, the actual electrolysis, to product separation, has to be considered.

### Carbon capture from point sources

For the electrochemical conversion of CO_2_ into valuable products, CO_2_ is required in high purity and must therefore be captured e. g. from the feedstock gas stream of a point source or directly from the atmosphere. The costs of capturing CO_2_ from power plants and chemical process exhausts using amine-based chemical absorption, which is mainly used for the CO_2_ capture from industrial flue gases, are currently at around 70 $ t^−1^. The amine-based solvent selectively absorbs CO_2_ via the formation of water-soluble salts (Zhang et al., 2020d). Via process optimization, a price of 40 $ t^−1^ could be reached, according to the techno economic analysis by Jiao, Sargent, and their coworkers (Jouny et al., 2018; Kibria et al., 2019; Raksajati et al., 2013, 2018). Common amine-based solvents are ethanolamine or *Mitsubishi*’s commercial KS-1 solvent. After CO_2_ absorption, the solvent is regenerated with water vapor. Via condensation of water, CO_2_ is then purified and further processed (Moioli et al., 2019; Rochelle, 2009). The drawback of the process is the high energy demand of the desorption process, as well as corrosiveness and amine loss during operation (Ho et al., 2009; Hüser et al., 2017; Rochelle, 2009). Therefore, Ho and colleagues calculated that the price of 70 $ t^−1^ could be reduced to 55 $ t^−1^ with the integration of waste heat into other processes (Ho et al., 2009). It is of particular importance that the solvents have the highest possible tolerance to SO_x_ and NO_x_ impurities, because solvent regeneration is a major contributor to operating costs. Furthermore, the solvents should exhibit low heats of reaction and evaporation rates, and be inexpensive to achieve deposition costs of about 37 $ t^−1^ (Raksajati et al., 2018). The use of KS-1 could lower the CO_2_ price only up to 46 $ t^−1^, which, however, is lower than for ethanolamine because of a lower regeneration energy demand. Besides ethanolamine and KS-1, other amine-based and amino acid-based solvents have been proposed (Chowdhury et al., 2013; Dubois and Thomas, 2012; Hüser et al., 2017; Moioli et al., 2019).

Alternatively to KS-1 and ethanolamine, it is possible to use weak alkali salts. Their application is characterized by lower regeneration costs, because less regeneration energy is required, and less degradation occurs. Using these solutions as an example, Ho and coworkers calculated a possible CO_2_ price of 30 $ t^−1^ for K_2_CO_3_. However, it is problematic that weak alkali salt solutions react less rapidly with CO_2_ than those containing amines. An increase in reaction rate can be achieved by adding additives such as boric acid or piperazine (Cullinane and Rochelle, 2004; Ho et al., 2009).

As other technologies to realize carbon capture at industrial facilities, oxy-combustion and calcium looping have been described (Vatopoulos and Tzimas, 2012; Yin and Yan, 2016). When pure oxygen instead of air is used for combustion, CO_2_ in high concentration is obtained for further utilization (Yin and Yan, 2016). The calcium looping process is based on the reversible reaction of CO_2_ with calcium oxide to calcium carbonate at temperatures between 600°C and 700°C, followed by the release of CO_2_ at temperatures above 900 °C (Martínez et al., 2011; Vatopoulos and Tzimas, 2012).

### Direct air capture

Capturing CO_2_ from point sources, such as waste gas streams, can reduce greenhouse gas emissions, whereas direct air capture (DAC) is a negative emission technology as it directly removes CO_2_ from the atmosphere. However, it has not been commercially realized yet and is challenging because of the low atmospheric concentrations of CO_2_. Assuming a CO_2_ capture capacity requirement of 0.98 Mt a^−1^, a techno-economic assessment issued in 2018 estimated a levelized cost of 94–232 $ t^−1^ CO_2_ (Keith et al., 2018). Potentially, DAC can involve capture using amine-functionalized solids, porous materials such as zeolites or MOFs, and the use of liquid alkaline sorbents (Kumar et al., 2015; Sanz-Pérez et al., 2016). When CO_2_ is adsorbed to solid sorbents, a lower regeneration temperature of 80°C–100°C is required to release it, and the remaining air can therefore be removed via a pressure drop by evacuation to some extent and/or insertion of steam (Fasihi et al., 2019; Kulkarni and Sholl, 2012; Sinha et al., 2017). A classification by Fasishi and colleagues divides the potential technologies into capture via aqueous solutions and regeneration at high temperatures (HT DAC), temperature swing adsorption (TSA) on solid sorbents and at low temperatures (LT DAC), and moisture swing adsorption (MSA) (Fasihi et al., 2019). For an LT DAC system case study, powered by a photovoltaic-wind-battery system in Morocco, the potential cost was calculated at 105 € t^−1^ and 60 € t^−1^ in 2030, depending on the possibility to use free waste heat from other facilities. A further reduction of the costs to 54 and 38 € t^−1^ in 2050 was proposed, based on reduced capital expenditures (CAPEX) with larger-scale implementation. The authors judge the LT DAC process as more promising, because of the possible use of excess waste heat (Fasihi et al., 2019). An example for the first attempts to implement DAC is the company *Carbon Engineering* with an LT DAC pilot plant with a capacity of 1 t d^−1^ (Keith et al., 2018). This plant absorbs CO_2_ with KOH in an air-liquid contractor and uses a CaO-calcium cycle for regeneration. The swiss-based company *Climeworks* developed an LT DAC process based on PSA on amine-modified porous granulates which reversibly bind CO_2_. The system is regenerated via pressure reduction and heating to 100°C (Climeworks, 2020; Christoph Gebald, Nicolas Repond, Jan Andre Wurzbacher). The company *Global Thermostat* is working on an LT DAC process based on amino-polymer sorbents, allowing for low cycle times of 30 min and regeneration at 85–94°C (Fasihi et al., 2019; Exxonmobil, 2019).

### Alternatives

In order to avoid the complex and expensive CO_2_ separation processes, there are approaches to operate CO_2_RR directly with industrial flue gases without previous CO_2_ capture. The challenges here are low CO_2_ concentrations, which lower the activity, and the contamination with other components. Oxygen can reduce the catalyst selectivity via the occurring ORR and substances like SO_x_ and NO_x_ act as catalyst poisons (Verma et al., 2019; Xu et al., 2020). Kenis and coworkers investigated the effect of CO_2_ concentration in the stream on the reduction to CO with a Ag catalyst. At 3.0 V cell potential, a diluted stream with 10% CO_2_ could still achieve a CO FE of over 80%. Switching from 100% to 10% CO_2_, the decrease in partial current density was below 45%. Lower voltages led to significantly reduced FEs (Kim et al., 2015). By using an ionomer layer containing hydrophilic nanopores which was bound with TiO_2_ nanoparticles, Sinton and colleagues could yield a C_2_ FE of 68% at 260 mA cm^−2^ from a simulated flue gas stream comprising 15% CO_2_ and 4% O_2_. The ionomer was successful in reducing O_2_ transport, and a pressure of up to 15 bar was applied to suppress the HER and increase the CO_2_ reduction activity (Xu et al., 2020). Oh and coworkers showed that the CO FE remains over 90% with an Au_25_ nanocluster catalyst reducing a 10% CO_2_ stream. They also demonstrated a 15.9% solar-to-CO conversion efficiency in an electrolyzer coupled to a Ga_0.5_In_0.5_P/GaAs photovoltaic cell (Kim et al., 2020a).

### Product separation

Not only carbon capture plays an important role in the techno-economic evaluation of the whole route of electrochemical CO_2_ reduction, but also the evaluation of the possible products. Because the CO_2_ reduction reaction can theoretically yield a variety of C_1_ to C_3_ chemicals, different target products are conceivable. To identify economically attractive products, the price, often normalized to the stored energy, and the market size is used (Jouny et al., 2018; Kibria et al., 2019). Table 5 provides an overview of the market prices of possible target products (Jouny et al., 2018). Based on these values, the authors selected methanol, ethylene, ethanol, and *n*-propanol as potential target products. However, among these products, methanol failed to yield a positive net present value (NPV) in the economic cost analysis (Jouny et al., 2018). For the analysis, the market value of pure CO as well as syngas, which is commonly used as an industrial feedstock mixture, was considered. Formic acid achieves the highest normalized market price, but shows low market volume, so its importance as a target product is limited. The market size for propanol is also small, but more efficient and consequently cheaper production could increase demand according to Jiao and coworkers (Jouny et al., 2018). Because of their high market size with a moderate normalized market price at the same time, Sargent and colleagues selected ethanol and ethylene as promising target products. Based on normalized prices, CO and formic acid would be the most attractive products, but would have a limited market (Kibria et al., 2019). However, the possibility of converting CO and formic acid to more valuable products also makes it an attractive target product.

**Table 5:** Market parameters of CO_2_ reduction products

Product	Global production [Mt year^−1^]	Market price [$ t^−1^]	Normalized market price [$ electron^−1^] ∗10^3^
methanol	110	580	3.1
ethanol	77	1,000	3.8
ethylene	140	1,300	3
*n*-propanol	0.2	1,430	4.8
CO (syngas)	150	60	0.8
CO	–	600	8
formic acid	0.6	740	16.1

Data according to (Jouny et al., 2018).

In addition to the market price, selectivities and process parameters also play a crucial role in the conversion of CO_2_ to value-added products. According to Masel and colleagues, the Faraday efficiency for multicarbon products should be at least 60% with a current density of at least 200 mA cm^−2^ (Masel et al., 2021). Furthermore, the use of KOH should be avoided, because it reacts with CO_2_ to form carbonate and furthermore causes the oxidation of copper catalysts when no reductive potential is applied. Potassium salts containing halides should also not be present in the electrolyte, as this would lead to the formation of gas products such as Cl_2_ or Br_2_ at the anode (Masel et al., 2021).

In addition, the processes required for product separation affect the overall costs of CO_2_ electrolysis. Although the separation processes have similar capital cost, liquid product separation has higher operational costs (Kibria et al., 2019). The estimated operational cost for the separation used by Sargent and coworkers were 10 $ t^−1^ for gas products via pressure swing adsorption, based on industrial biogas separation, and 60 $ t^−1^ for liquid product separation via distillation with a minimum input of 10 wt % liquid product. Possible further process steps for gas products, like compression for transport, were not considered (Dahmus and Gutowski, 2007; Greenblatt et al., 2018; Jouny et al., 2018; Kibria et al., 2019).

### Capital & operating costs of the electrolyzer

Apart from the target product, the capital and operating costs of the utilized electrolyzer system, as well as additional process steps, contribute to the economic viability of the overall technology. Based on similar costs for PEM water electrolyzers, the electrolyzer cost was estimated at 5,000–15,000 $ m^−2^ by Sargent and colleagues, whereas Jiao and coworkers estimated 920 $ m^−2^ for the optimistic case of their model (Jouny et al., 2018; Kibria et al., 2019). The operational cost of the electrolyzer is largely dependent on electricity costs, more so for products that require a higher number of reduction steps. The required electricity can be reduced by achieving higher energy efficiencies of the system (Kibria et al., 2019). For the calculation of their techno-economic models, the estimated electricity cost used by Sargent and coworkers was two cents KWh^−1^, based on estimates of the US Department of Energy for 2030 (Solar Energy Technologies office, 2020; Kibria et al., 2019). Jiao and colleagues assumed costs of three cents KWh^−1^ based on estimations for 2030 for the optimistic case (Haegel et al., 2017; Jouny et al., 2018).

To ensure profitable production cost for a desired product, the multicomponent system consisting of an electrochemical cell and catalyst must meet certain performance targets and should be considered one entity.

(1) The reaction rate, in terms of the electrochemical reduction, is defined via the current density. Required target values for current density and FE are related to the capital cost, because lower values require a larger electrolyzer size to reach the target rate of production. In the techno-economic model of Sargent and colleagues, a reduction of the current density from 300 mA cm^−2^ to 100 mA cm^−2^ would raise the price by more than 500 $ t^−1^ of ethanol (Kibria et al., 2019). Overall, industrial CO_2_ reduction processes should aim at current densities of at least 300 mA cm^−2^ (Jouny et al., 2018; Kibria et al., 2019).
(2) Another important parameter is the cell voltage, which determines the energy efficiency of the electrolyzer. Thus, a reduced cell voltage lowers the power requirement. This is especially relevant for products, which need a high number of reduction steps. Sargent and coworkers showed that with a cell voltage of 1.8 V, several products would become economically feasible with 90% FE (Kibria et al., 2019). Jiao and colleagues chose a cell voltage of 2 V for the optimist case in their techno-economic model (Jouny et al., 2018).
(3) Furthermore, the system lifetime is a factor for the overall economic viability. Sargent and colleagues proposed a lifetime of 80,000 h as desirable, based on values reached by industrial water electrolyzers (Kibria et al., 2019). Jiao and coworkers assumed a 20-year lifetime with 350 days per year operating time (Jouny et al., 2018). Perez-Ramirez and colleagues postulated a much lower lifetime of at least 5,000 h as requirement (Martín et al., 2015). Spurgeon and coworkers assumed a system lifetime of 20 years with costs for replacement of the major components after 7 and 14 years (Spurgeon and Kumar, 2018).

Table 6 shows the proposed key performance indicators that could yield a positive net present value in the techno-economic studies of Sargent, Jiao, and their coworkers.

**Table 6:** Electrolyzer KPI targets

	(Jouny et al., 2018)	(Kibria et al., 2019)
Faradaic efficiency [%]	90	80–90
Current density [mA cm-2]	300	>300
Cell voltage [V]	2	<1.8
Lifetime [h]	168,000	>80,000

In their techno-economic evaluation of low-temperature electrochemical reduction of CO_2_ to CO, Masel and colleagues identified the lack of alternatives for iridium as anode catalyst and the necessity of lowering the cell voltage while avoiding the use of wearing materials (e.g. KOH or additives) as key factors that impede application on an industrial scale. For the production of formate, there is a lack of sufficiently selective and stable catalysts, while C_2+_ products cannot be obtained yet with adequate current densities or FEs without the use of KOH or halides as electrolytes (Masel et al., 2021).

### Tandem processes

Although the normalized market price of CO is lower compared to other CO_2_RR products, reduction of CO_2_ to CO with in-line processing to further value-added products may be a profitable pathway (Haas et al., 2018; Krause et al., 2020; Schmidt et al., 2018). High-temperature co-electrolysis of CO_2_ and water to syngas in SOECs is nowadays comparably close to reaching industrially relevant and profitable performance. In a direct comparison of an alkaline liquid-phase flow cell, a neutral MEA, and a tandem CO_2_-CO-ethylene process, Sargent and colleagues concluded that the one-step processes suffer from low energy efficiency and CO_2_ losses because of crossover and carbonate formation, markedly contributing to the system costs. However, avoiding carbonate formation by utilizing the two-step tandem process leads to possible profitability at viable system parameters. Provided that the first step, the electrolysis of CO_2_ to CO in a SOEC, reaches an energy efficiency of 80%, and in the second step ethylene can be obtained with an energy efficiency of 40%, a price of 1,000 $ t^−1^ could be achieved at electricity costs of 0.02 $ kWh^−1^. Already in 2010, Fu and coworkers modeled the combination of CO_2_/H_2_O co-electrolysis in a SOEC, followed by Fischer-Tropsch process to liquid fuels. They pointed out the beneficial carbon balance as a benefit in comparison to gas-to-liquid and coal-to-liquid processes, but stated the importance of low-cost renewable electricity and concentrated CO_2_ feed for the profitability (Fu et al., 2010). As additional factors, Bao and coworkers identified extended lifetimes (>50,000 h), lower overpotentials, and a suitable successive conversion of the produced syngas as crucial parameters for market maturity (Song et al., 2019). The necessity of enhanced lifetimes was confirmed by Desideri and colleagues when examining CO_2_/water co-electrolysis with subsequent conversion to methanol. Furthermore, they found that the stack costs still have to be reduced and that the supply of excess renewable energy to the system must be stable to maintain reasonable payback times. Benefits of the technology, however, are its high energy and carbon-conversion efficiencies of 72 and 93.6%, respectively, and good heat integration properties between high-temperature electrolysis and methanol production (Zhang and Desideri, 2020). As energy efficiencies of >80% are already reached in commercially available SOECs, CO-to-ethylene electrolyzers with energy efficiencies of 30% are already reported, and ethylene is nowadays traded for 800–1,200 $ t^−1^, the economic perspectives of the tandem process are encouraging (Sisler et al., 2021).

## Outlook & trends

CO_2_ electrolysis is an emerging technology, which will most likely contribute to both energy storage and base chemicals supply in the future. Particularly the production of syngas via high-temperature co-electrolysis of CO_2_ and water is already possible at industrial scale and close to being economically competitive. Nevertheless, there are still challenges that have to be solved to allow for full industrial implementation. A majority of research data concerning CO_2_ electrolysis refers to purified CO_2_ feedstocks. However, the production of high-purity CO_2_ is cost-intensive and it should be kept in mind to develop catalyst and reactor systems that are able to work with either impure or diluted CO_2_ streams. In addition to that, there is still a general lack of sufficiently robust and selective electrocatalysts for CO_2_ reduction to individual products. Product separation is another complex and costly procedure, which is why it is desirable to obtain highly concentrated single products. In case of CO and formic acid, this criterion can already be met at a large scale, but the production of C_2+_ products has to be improved in this regard. Furthermore, most of the catalysts employed in research are either based on precious metals or require complex manufacturing procedures, which make them cost-intensive and insufficiently abundant for industrial implementation. It has to stay in mind that there is also an urgent need for alternative anode catalysts under acidic conditions as the state-of-the-art material iridium does not meet those criteria. Gas-diffusion electrodes became the predominant electrode design because of overcoming the mass-transport limitation of CO_2_. Although significant advances were made in recent years, some unsolved issues remain. Research efforts mostly focus on carbon-based and PTFE-based GDLs. Although carbon-based GDLs are not suitable for upscaling because of their high costs and insufficient mechanical stability, PTFE membranes cannot deliver through-plane electrical conductivity and are therefore not suitable for stacking. Current research is already focusing on alternative materials, which combine electric conductivity with sufficient gas permeability and hydrophobicity to create highly functional GDLs. Furthermore, the long-term water management at the CL presents a challenge. In many cases, flooding causes significant performance losses over time, leading to favored HER, and consequently, clogging because of carbonate formation. To improve water management, zero-gap PEM cells were developed, where water is no longer supplied via liquid electrolytes but a humidified CO_2_ feed instead. Another advantage of the renunciation of liquid electrolytes is the elimination of wear materials. Alternatively, the electrolysis of supercritical CO_2_ might also be an attractive way to overcome CO_2_ mass transport limitations as well as challenges of water management and product separation. Furthermore, CO_2_ itself serves as a solvent in this technology. Certainly, the technical requirements for this technology are increased and efforts must be made to avoid product reoxidation. Another way to enhance CO_2_ availability is the application of differential pressure on the gas side, which enables operation at higher current densities compared to atmospheric conditions. Challenges for the application of this technique are increasing mechanical stress on the membrane and enhanced CO_2_ crossover. However, the success of CO_2_ electrolysis in most cases depends on conductive membranes and ionomers. Those highly specialized materials are to date hardly available on a sufficient scale, at moderate costs and consistent quality. Furthermore, a balance between mechanical robustness and sufficiently low resistance is still to be found to lower overall cell voltage. Future research efforts should therefore also focus on the development of highly conductive polymers with a scalable synthesis. It is noticeable that because of environmental concerns, fluorine-free ionomers should be developed. In conclusion, products of CO_2_ electrolysis are, apart from CO, not yet economically competitive. It is indeed important to gain mechanistic insights into the complex CO_2_RR by fine-tuning sophisticated catalysts and ionomers. However, to enable the economic future of these processes, the focus of research must eventually shift to large-scale applicable and affordable materials. Therefore, close and interdisciplinary collaboration between academia and industry is necessary to ensure technological progress in the general field of electrolysis (Siegmund et al., 2021). Time will tell if science overcomes this challenge and which potential products of CO_2_RR will prospectively be produced via electrochemical methods.

### Limitations of the study

Because of the tremendous research progress and accordingly high number of publications on the topic, this review does not include every single study on the technical aspects of electrochemical CO_2_ reduction. However, no specific author was excluded intentionally. With the division of the manuscript into two parts, we try to cover as many aspects of the topic as possible. The focus of the catalytic considerations thereby lies on the production of multicarbon alcohols though. Other products of electrochemical CO_2_R require different catalysts and reaction conditions than the ones presented.
